# Asymmetric desymmetrization of *meso*-diols by *C*_2_-symmetric chiral 4-pyrrolidinopyridines

**DOI:** 10.3762/bjoc.8.203

**Published:** 2012-10-17

**Authors:** Hartmut Schedel, Keizo Kan, Yoshihiro Ueda, Kenji Mishiro, Keisuke Yoshida, Takumi Furuta, Takeo Kawabata

**Affiliations:** 1 Institute for Chemical Research, Kyoto University, Uji, Kyoto 611-0011, Japan

**Keywords:** acylation, desymmetrization, hydrogen bond, *meso-*diol, nucleophilic catalyst, organocatalysis

## Abstract

In this work we developed *C*_2_-symmetric chiral nucleophilic catalysts which possess a pyrrolidinopyridine framework as a catalytic site. Some of these organocatalysts effectively promoted asymmetric desymmetrization of *meso*-diols via enantioselective acylation.

## Introduction

**Figure 1:**
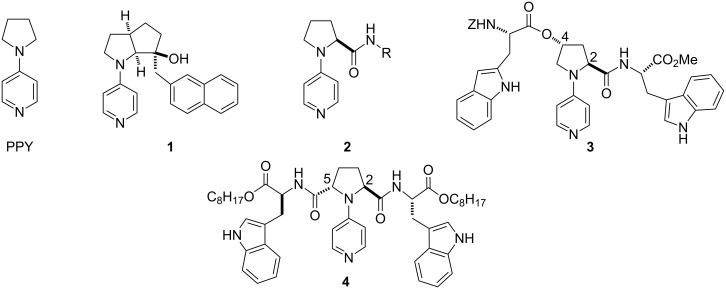
Chiral PPY catalysts.

Since the pioneering discovery of a catalyst for enantioselective acylation by Vedejs [1], numerous efforts have been devoted to the development of catalysts for enantioselective acylation [2–3]. We have focused on the development of chiral nucleophilic catalysts possessing a pyrrolidinopyridine (PPY) framework as a catalytic site because PPY has been known to be one of the most powerful catalysts for the acylation of alcohols [4–7]. The salient feature of our catalyst design is to introduce chiral elements far from the catalytically active pyridine nitrogen as shown in Figure 1 [8–18]. These catalysts are expected to show high catalytic activity because the introduction of substituents close to the pyridine nitrogen has been known to result in the significant decrease of the catalytic activity [19]. Catalyst **1** was demonstrated to be effective for the kinetic resolution of racemic diols (*s*: up to 12) [8] and amino alcohol derivatives (*s*: up to 54) [9]. Catalyst **2**, readily prepared from L-proline, could be employed for the kinetic resolution of amino alcohol derivatives (*s*: up to 11) [10]. Chiral PPY catalysts with dual functional side chains at C(2) and C(4) of the pyrrolidine ring such as **3** were prepared from *trans-*4-hydroxy-L-proline. These catalysts were found to be moderately effective for the asymmetric desymmetrization of *meso*-diols [11]. *C*_2_-Symmetric PPY-catalyst **4** was found to be effective for the chemo- and regioselective acylation of carbohydrates [12,14,16] and the chemoselective monoacylation of linear diols [17]. Here, we report the asymmetric desymmtrization of *meso*-diols by *C*_2_-symmetric PPY catalysts [20]. The effects of the functional side chains at C(2) and C(5) on the efficiency of the asymmetric desymmetrization are discussed. Some of the results shown here have already been appeared in the patent JP2005132746.

## Results and Discussion

### Asymmetric desymmetrization of *meso*-1,2-cyclohexanediol

**Scheme 1:**
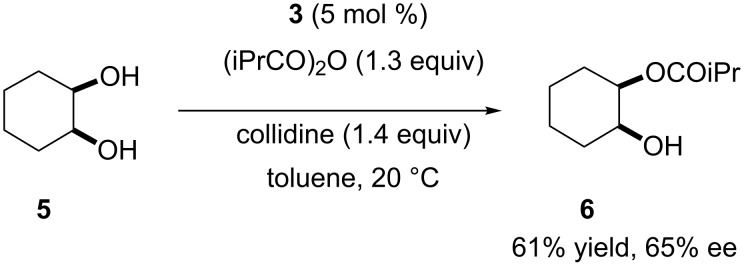
Asymmetric desymmetrization of **5** with catalyst **3**.

**Scheme 2:**
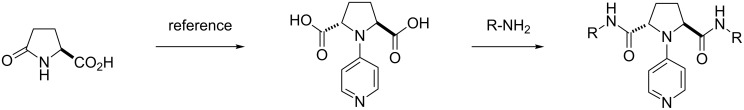
Preparation of a small library of chiral *C*_2_-symmetric PPY catalysts (reference, see [12]).

The asymmetric desymmetrization of *meso-*diols via organocatalytic enantioselective acylation has been extensively studied [1,21–38]. We have reported the asymmetric desymmetrization of *meso*-1,2-cyclohexanediol (**5**) by catalytic enantioselective acylation with catalyst **3** (Scheme 1) [11]. Among various chiral PPY catalysts with dual functional side chains at C(2) and C(4) of the pyrrolidine ring, catalyst **3** was found to be most effective for the asymmetric desymmetrization (Scheme 1). However, the enantioselectivity of the asymmetric desymmetrization was far from being sufficient. Molecular modeling of the related catalysts indicated that a *C*_2_-symmetric PPY catalyst with functional side chains at C(2) and C(5) might be better suited for this purpose [12–13]. Based on these background, we have prepared various *C*_2_-symmetric chiral PPY-catalysts according to Scheme 2 [12] and employed them for asymmetric desymmetrization of *meso*-1,2-cyclohexanediol (**5**) [20].

We first examined chiral PPY catalyst **8a** with an L-tryptophan side chain, which was disclosed to be an excellent catalyst for the regioselective acylation of glycopyranoses [12]. Reaction of **5** with isobutyric anhydride (1.3 equiv) in the presence of 5 mol % of **8a** in chloroform at 20 °C gave monoacylate **6** in 73% ee and 85% yield with concomitant formation of 15% of diacylate **7** (Table 1, entry 1). Catalyst **8b** with a D-tryptophan side chain gave a lower enantioselectivity (54% ee, Table 1, entry 2). The hydroxy group at the (*R*)-chiral center was preferentially acylated in both cases. Since both catalysts **8a** with an L-tryptophan side chain and **8b** with a D-tryptophan side chain gave (1*R*,2*S*)-**6** [8] by asymmetric desymmetrization of **5**, catalysts with achiral side chains were then examined. The acylation of **5** with catalyst **9** which possesses a tryptamine moiety gave **6** in 72% ee and 74% yield (Table 1, entry 3). Catalyst **10** with a glycine moiety gave **6** in 71% ee and 72% yield on treatment of **5** (Table 1, entry 4). Monoacylate **6** was also obtained in 87% ee and 75% yield by acylation of **5** with catalyst **11** which possesses a simple *n*-hexyl side chain (Table 1, entry 5). Catalysts with chiral side chains, **12a**, **12b**, **13**, and **14**, possessing L-β-phenylalanine, D-β-phenylalanine, L-valine, or L-leucine moiety, respectively, also gave **6** in 54–83% ee and 70–77% yields via acylative asymmetric desymmetrization of **5** with isobutyric anhydride (Table 1, entries 6–9). In each case, (1*R*,2*S*)-**6** was preferentially obtained. These results indicate that the functionality and chirality of catalyst side chains do not affect the absolute configuration of the monoacylate obtained by the asymmetric desymmetrization while they influence the extent of the enantioselectivity. Accordingly, the configuration of the stereocenters in C(2) and C(5) position bearing the amide substituents appears to have decisive effects on the stereochemical course of the asymmetric desymmetrization.

**Table 1:** Effects of the catalysts’ side chains on the asymmetric desymmetrization of *meso*-1,2-cyclohexanediol (**5**).^a^

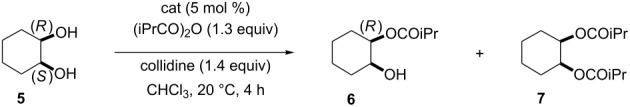			

Entry	Catalyst^b^	**6**:**7**:Recovery of **5** (%)^c^	ee of **6** (%)^d,e^

1	**8a**	85:15:0	73
2	**8b**	70:18:12	54
3	**9**	74:16:10	72
4	**10**	72:19:9	71
5	**11**	75:23:3	87
6	**12a**	70:25:5	74
7	**12b**	77:14:9	83
8	**13**	76:18:6	54
9	**14**	75:20:5	81

^a^Reactions were run at a substrate concentration of 0.2 M. ^b^Structures of catalysts:
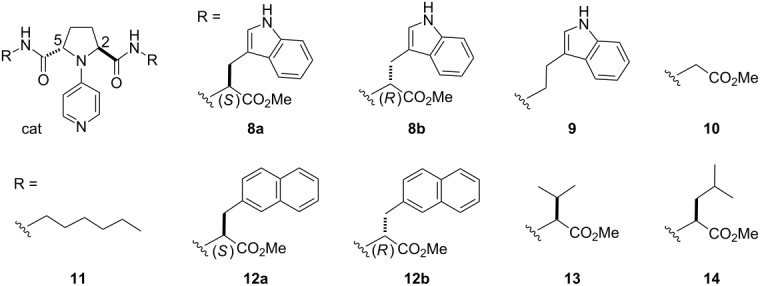
. ^c^Yields determined by ^1^H NMR with dibenzyl ether as an internal standard. ^d^Determined by GC analysis with a chiral stationary phase, beta-DEX 225. ^e^(1*R*,2*S*)-Isomer was obtained in each case.

**Figure 2:**
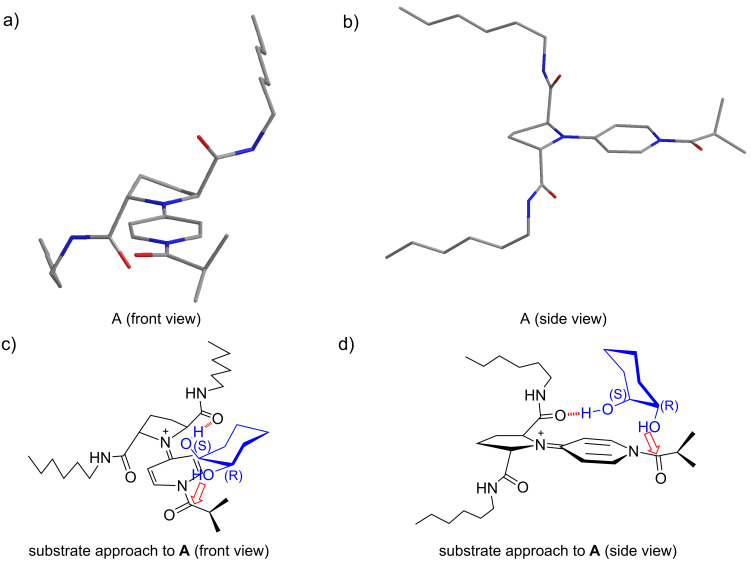
A hypothetical model for the transition-state assembly of the asymmetric acylation of **5** promoted by catalyst **11**. Front view a) and side view b) of the calculated structure of acylpyridinium ion **A**. Front view c) and side view d) of the possible modes for the substrate approach to **A**.

**Figure 3:**
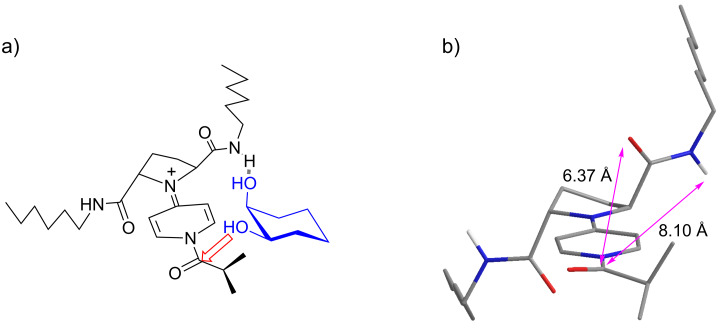
An alternative model for the transition state assembly of the asymmetric acylation of **5** promoted by catalyst **11**. a) A possible mode of the substrate approach to acylpyridinium ion **A**, and b) the calculated structure of **A**.

Since catalyst **12b** showed relatively high enantioselectivity (83% ee) and mono/diacylation ratio (77:14), we next investigated the solvent effects of the asymmetric desymmetrization of **5** with isobutyric anhydride in the presence of catalyst **12b** (Table 2, entries 1–5). A clear relationship between the enantioselectivity and the solvent polarity was observed: The lower the polarity of the solvent, the higher the enantioselectivity. This suggests that the hydrogen-bonding interaction between the catalyst and the substrate may be involved in the transition state of the enantioselective acylation (Figure 2 and Figure 3). The temperature effects (lower temperature) of the asymmetric desymmetrization employing catalysts **14** and **11** were examined because both of the catalysts show high solubility in chloroform at low temperatures (Table 2, entries 6–9). Both enantioselectivity (87% ee) and mono/diacylation ratio (85:6) of the acylation of **5** catalyzed by **14** at −40 °C were improved compared with those in the corresponding reaction at 20 °C (Table 2, entry 6 vs entry 7). Similarly, the efficiency of the asymmetric desymmetrization of **5** catalyzed by **11** was improved by conducting the reaction at −40 °C (Table 2, entry 8 vs entry 9). Monoacylate **6** was obtained in 88% ee and 92% yield by treatment of **5** with isobutyric anhydride in the presence of 5 mol % of **11** at −40 °C.

**Table 2:** Effects of solvents and temperature on the asymmetric desymmetrization of **5**.^a^

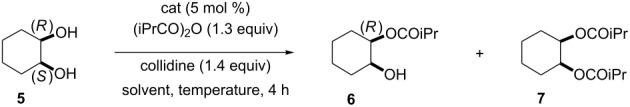					

Entry	Catalyst	Solvent	Temp. (°C)	**6**:**7**:recovery of **5** (%)^b^	ee of **6** (%)^c,d^

1	**12b**	CCl_4_	20 °C	75:20:5	93
2	**12b**	toluene	20 °C	75:19:6	91
3	**12b**	CHCl_3_	20 °C	77:14:9	83
4	**12b**	THF	20 °C	57:28:15	51
5	**12b**	CH_3_CN	20 °C	69:23:8	34
6	**14**	CHCl_3_	20 °C	75:20:5	81
7^e^	**14**	CHCl_3_	**−**40 °C	85:6:9	87
8	**11**	CHCl_3_	20 °C	75:23:2	87
9^e^	**11**	CHCl_3_	**−**40 °C	92:5:3	88

^a^Reactions were run at a substrate concentration of 0.2 M. ^b^Yields determined by ^1^H NMR with dibenzyl ether as an internal standard. ^c^Determined by GC analysis with a chiral stationary phase, beta-DEX 225. ^d^(1*R*,2*S*)-Isomer was obtained in each case. ^e^Run for 24 h.

**Scheme 3:**
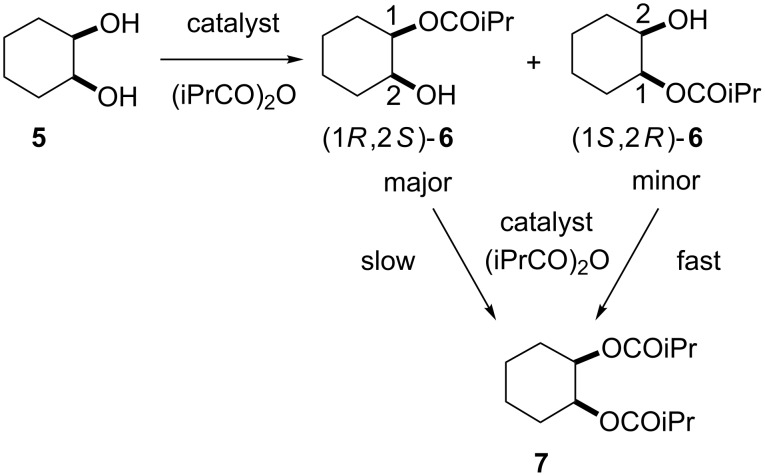
Amplification of enantiomeric purity of the major enantiomer produced at the step of asymmetric desymmetrization of the *meso*-substrate by the following kinetic resolution with the same catalyst.

Further optimization of the asymmetric desymmetrization of **5** with catalyst **11** was examined at 20 °C (Table 3). The use of only 0.5 mol % of catalyst was found to be still effective in the asymmetric desymmetrization of **5** to give **6** in 90% ee and 76% yield (Table 3, entry 2) [20]. Further decrease in the amount of the catalyst to 0.05 mol % resulted in a lower enantioselectivity (74% ee) and in lower yield (66%) (Table 3, entry 3). The use of a less amount (1.0 equiv) of the anhydride in the presence of 5 mol % of **11** improved the mono/diacylation ratio (84:7), while the enantioselectivity was decreased (81% ee, Table 3, entry 1 vs entry 4). On the other hand, the use of an excess amount (1.6 equiv) of the anhydride resulted in the highest enantioselectivity (98% ee) in compensation for the low yield (59%) for monoacylation (Table 3, entry 5) [20]. The increase in the amount of diacylate **7** is associated with the higher ee of monoacylate **6** (Table 3, entries 1, 4 and 5). This suggests that the ee of monoacylate **6** would be amplified by the second acylation step, i.e., acylative kinetic resolution of enantioenriched monoacylate **6** produced by the asymmetric desymmetrization of the *meso-*substrate (Scheme 3).

**Table 3:** Optimization of the asymmetric desymmetrization of **5** with catalyst **11**.^a^

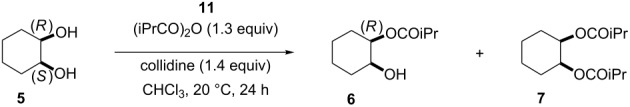				

Entry	Mol % of **11**	Equiv of (iPrCO)_2_O	**6**:**7**:Recovery of **5** (%)^b^	ee of **6** (%)^c,d^

1	5	1.3	75:23:2	87
2^e^	0.5	1.3	76:20:3	90
3	0.05	1.3	66:10:24	74
4	5	1.0	84: 7:4	81
5^e,f^	5	1.6	59:41:0	98

^a^Reactions were run at a substrate concentration of 0.2 M. ^b^Yields determined by ^1^H NMR with dibenzyl ether as an internal standard. ^c^Determined by GC analysis with a chiral stationary phase, beta-DEX 225. ^d^(1*R*,2*S*)-Isomer was obtained in each case. ^e^Data quoted from reference [20]. ^f^1.7 Equiv of collidine were used.

**Scheme 4:**
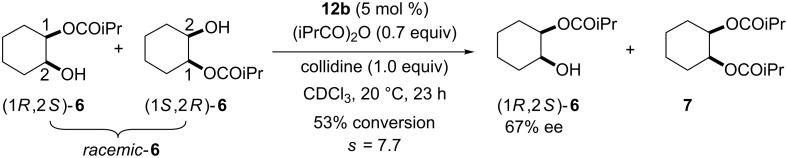
Acylative kinetic resolution of *racemic*-**6** with catalyst **12b**.

To confirm this issue, kinetic resolution of *racemic*-**6** was performed with catalyst **12b** because **12b** is almost as effective as **11** in the asymmetric desymmetrization of **5** (Table 1, entry 5 and entry 7). Treatment of *rac*-**6** with 0.7 equiv of isobutyric anhydride in the presence of 5 mol % of **12b** gave (1*R*,2*S*)-**6** in 67% ee at 53% conversion (Scheme 4). This clearly indicates that the (1*S*,2*R*)-isomer reacts faster than the (1*R*,2*S*)-isomer in the acylation catalyzed by **12b**.

The results in Table 1 suggest that the amide carbonyl groups at C(2) and C(5) of the pyrrolidine ring of the catalysts **8**–**14** would play the key role in asymmetric acylation. This is due to the fact that the amide carbonyl group is the common structural subunit among these chiral PPY catalysts. We chose catalyst **11**, which possesses the simplest *n*-hexyl amide side chain, and examined the effect of the secondary amide linkage by comparing the performance of the asymmetric desymmetrization with that shown by the analogous catalysts possessing the corresponding tertiary amide- or ester linkage, **15** or **16**, respectively (Table 4, entries 1–3). Much diminished enantioselectivity (13% ee) was observed in the asymmetric desymmetrization of **5** with isobutyric anhydride in the presence of catalyst **15** with the tertiary amide linkage (Table 4, entry 2). Similarly, catalyst **16** with the ester linkage was found to be far less effective (13% ee) than **11** (Table 4, entry 3) in the asymmetric acylation. These results indicate that the secondary amide linkage in **11** is essential for the high efficiency of the asymmetric acylation. The superior property of **11** compared to **16** as an asymmetric acylation catalyst could be ascribed to the stronger Lewis basicity of the amide carbonyl group than that of the ester carbonyl group (donor number of amides > donor number of esters). However, the reasons for the poorer efficiency of catalyst **15** with a tertiary amide linkage compared with catalyst **11** with a secondary amide linkage are unclear (see also Figure 3). We then examined the effects of the *C*_2_-symmetric structure of catalysts **11** and **12a** by comparing the corresponding mono-functionalized chiral PPY catalysts **17** and **18** [10], respectively (Table 4, entry 4 and entry 5). Catalyst **17** was found to be slightly less effective than **11** in the asymmetric desymmetrization of **5** to give the monoacylate in 76% ee (Table 4, entry 4). Catalyst **18** gave monoacylate **6** in diminished ee (41% ee) in the acylative desymmetrization of **5** (Table 4, entry 5 vs entry 6). The corresponding (1*R*,2*S*)-**6** was obtained in each case. These results imply that a *C*_2_-symmetric structure in catalysts is responsible for the higher efficiency in the asymmetric acylation.

**Table 4:** Effects of side chain linkage and *C*_2_-symmetric structure of catalysts on the asymmetric desymmetrization of **5**.^a^

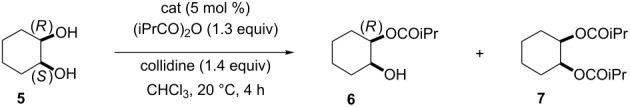			

Entry	Catalyst^b^	**6**:**7**:Recovery of **5**^c^ (%)	ee of **6** (%)^d,e^

1	**11**	75:23:3	87
2	**15**	79:15:6	13
3	**16**	65:24:11	13
4	**17**	70:26:4	76
5	**18**	61:34:5	41
6	**12a**	70:25:5	74

^a^Reactions were run at a substrate concentration of 0.2 M. ^b^Structures of catalysts
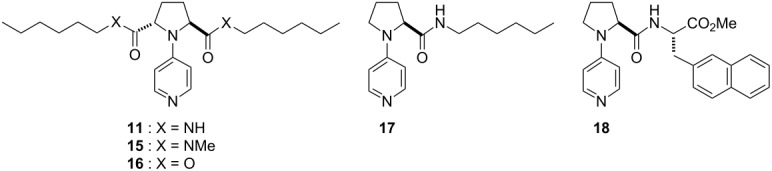
. ^c^Yields determined by ^1^H NMR with dibenzyl ether as an internal standard. ^d^Determined by GC analysis with a chiral stationary phase, beta-DEX 225. ^e^(1*R*,2*S*)-Isomer was obtained in each case.

### Asymmetric desymmetrization of *meso*-1,3-cyclohexanediol

We have reported that catalyst **3** promoted the acylative asymmetric desymmetrization of *meso*-1,3-cyclohexanediol (**19**) to give **20** in 52% ee and 48% yield [11]. Here, *C*_2_-symmetric chiral PPY catalysts were examined for this asymmetric transformation (Table 5). Treatment of **19** with isobutyric anhydride in the presence of catalyst **4** at 0 °C gave monoacylate **20** in 48% ee and 66% yield (Table 5, entry 1). The corresponding reaction at −40 °C did not improve the enantioselectivity (Table 5, entry 2). The attempted asymmetric desymmetrization of **19** at 20 °C promoted by catalysts **11**, **12a**, **12b**, and **14** resulted in the formation of the monoacylate in 19–31% ee and 48–69% yield (Table 5, entries 3, 5–7). Lowering the temperature of acylation of **19** in the presence of **11** did not improve the enantioselectivity (Table 5, entry 3 vs entry 4). The lack of temperature effects may indicate that the hydrogen-bonding interaction between the catalyst and the substrate may not significantly be involved in the process of enantioselective acylation of **19** in the presence of catalysts **4** and **11**.

**Table 5:** The asymmetric desymmetrization of *meso*-1,3-cyclohexanediol (**19**) with *C*_2_-symmetric chiral PPY catalysts.^a^

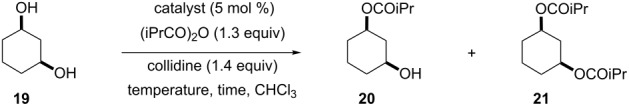					

Entry	Catalyst	Temp. (°C)	Time (h)	**20**:**21**:Recovery of **19** (%)^b^	ee of **20** (%)^c,d^

1	**4**	0	12	66:15:17	48
2	**4**	–40	48	26: 2:72	50
3	**11**	20	4	69:24:7	31
4	**11**	–40	48	44:10:44	25
5	**12a**	20	4	48:43:9	27
6	**12b**	20	4	49:30:21	20
7	**14**	20	4	62:33:6	19

^a^Reactions were run at a substrate concentration of 0.2 M. ^b^Yields determined by ^1^H NMR with dibenzyl ether as an internal standard. ^c^Determined by GC analysis with a chiral stationary phase, beta-DEX 225. ^d^The absolute configuration of **20** was not determined.

### Asymmetric desymmetrization of *meso*-2,3-butanediol and *meso*-hydrobenzoin

The asymmetric desymmetrization of *meso*-2,3-butanediol (**22a**) and *meso*-hydrobenzoin (**22b**) were examined (Table 6). Treatment of **22a** with isobutyric anhydride in the presence of 5 mol % of catalyst **8a** at 20 °C for 4 h gave monoacylate **23a** in 53% ee and 78% yield (Table 6, entry 1). Catalysts **10**, **11**, **12b**, and **14** were also examined for asymmetric desymmetrization of **22a** (Table 6, entries 2–8). These catalysts are almost equally effective in the asymmetric desymmetrization of **22a** at 20 °C to give monoacylate **23a** in 57–66% ee and 72–78% yield (Table 6, entries 2, 3, 5, and 6). As observed in the asymmetric desymmetrization of **5**, the acylation of **22a** at a lower temperature gave better selectivity. The catalytic enantioselective acylation of **22a** with isobutyric anhydride in the presence of **11** or **14** at −60 to −65 °C gave monoacylate **23a** in 87% ee (72% yield) or 92% ee (61% yield), respectively (Table 6, entries 4 and 8) [20] . The higher enantioselectivity was found to be associated with the higher mono/diacylation ratio in the asymmetric acylation of **22a** promoted by **14** (Table 6, entries 6–8). (Notice: Enantioenriched **23a** gradually undergoes partial racemization when it is kept as a CHCl_3_ solution probably via intramolecular acyl migration: e.g*.*, from 88% ee to 71% ee after 168 h.) The asymmetric desymmetrization of *meso*-hydrobenzoin (**22b**) was examined. Treatment of **22b** with isobutyric anhydride in the presence of catalysts **8a**, **8b**, and **12b** at 20 °C gave **23b** in 19–40% ee in 54–64% yield (Table 6, entries 9–11). Significant amounts of the diacylate were also formed (22–25% yield) together with the recovery of the unreacted material (11–21%). In these transformations, the low enantioselectivity is associated with the low mono/diacylation ratio, which was also observed in the asymmetric desymmetrization of *meso*-1,3-cyclohexanediol (**19**, Table 5). The asymmetric desymmetrization of *meso*-1,2-cyclopentanediol (**22c**) was also examined using catalysts **4** and **25** [18], the corresponding octyl ester analogues of **8a** and **12a**, respectively (Table 6, entries 12–14). The acylation of **22c** with isobutyric anhydride in the presence of **4** in chloroform at −20 °C gave monoacylate **23c** as a racemate in 85% yield (Table 6, entry 12). Similarly, racemic **23c** was obtained by the reaction of **22c** with isobutyric anhydride in the presence of **25**, either in chloroform or in toluene (Table 6, entries 13 and 14).

**Table 6:** The asymmetric desymmetrization of *meso*-2,3-butanediol (**22a**), *meso*-hydrobenzoin (**22b**) and *meso*-1,2-cyclopentanediol (**22c**).^a^

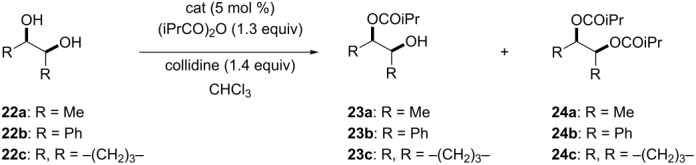						

Entry	Substrate	Catalyst^b^	Temp. (°C)	Time (h)	**23**:**24**:Recovery of **22** (%)^c^	ee of **23** (%)

1	**22a**	**8a**	20	4	78:11:11	53^d,e^
2	**22a**	**10**	20	4	77:16:7	62^d,e^
3	**22a**	**11**	20	4	78:13:9	66^d,e^
4^f^	**22a**	**11**	−60	24	72:7:21	87^d,e^
5	**22a**	**12b**	20	4	72:6:22	61^d,e^
6	**22a**	**14**	20	24	73:18:9	57^d,e^
7	**22a**	**14**	−40	24	82:4:14	85^d,e^
8^f^	**22a**	**14**	−65	24	61:<1:39	92^d,e^
9	**22b**	**8a**	20	4	64:25:11	40^g,h^
10	**22b**	**8b**	20	4	63:22:15	23^g,h^
11	**22b**	**12b**	20	4	54:25:21	19^g,h^
12	**22c**	**4**	−20	6	85:12:3	~0^i^
13	**22c**	**25**	−20	4	73:27:0	~0^i^
14^j^	**22c**	**25**	−20	4	67:33:0	~0^i^

^a^Reactions were run at a substrate concentration of 0.2 M. ^b^Structure of catalyst **25**:
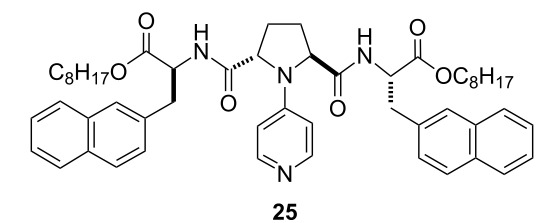
^c^Yields determined by ^1^H NMR with dibenzyl ether as an internal standard. ^d^Ee was determined by GC analysis with a chiral stationary phase, beta-DEX 225. ^e^(2*R*,3*S*)-Isomer was obtained. ^f^Data quoted from reference [20]. ^g^Ee was determined by HPLC analysis with a chiral stationary phase, Chiralcel OJ (iPrOH:hexane = 5:95, flow 0.5 mL min^−1^, *t*_R_ = 35, 51 min). ^h^The absolute configuration was not determined. ^i^Ee of the corresponding benzoate, which was determined by HPLC analysis with a chiral stationary phase, Chiralcel AS (iPrOH/hexane 1:99, flow 0.2 mL min^−1^, *t*_R_ = 43, 49 min). ^j^Run in toluene.

### Mechanistic implication

Several characteristic phenomena were observed in the asymmetric desymmetrization of *meso*-diols promoted by *C*_2_-symmetric chiral PPY catalysts. (1) Substrate specificity: *Meso-*1,2-cyclohexanediol (**5**) and *meso-*2,3-butanediol (**22a**) (matched substrates) gave high enantioselectivity, while *meso*-1,3-cyclohexanediol (**19**), *meso*-hydrobenzoin (**22b**), and *meso*-1,2-cyclopentanediol (**22c**) (mismatched substrates) gave poor enantioselectivity in the asymmetric desymmetrization. (2) A higher enantioselectivity was observed in the reactions of matched substrate **5** in the solvents of the lower polarity (Table 2, entries 1–5). (3) A higher enantioselectivity and a higher mono/diacylation ratio were observed in the acylation of the matched substrates at the lower temperatures (Table 2, entries 6–9; Table 6, entries 3, 4, 6–8). These phenomena suggest that the enantioselective acylation of the matched substrates proceeds in an accelerative manner via hydrogen-bonding interaction between the catalyst and the substrate. A possible model for the transition state assembly for the enantioselective acylation of *meso*-1,2-cyclohexanediol (**5**) catalyzed by **11** is shown in Figure 2. A chiral acylpyridinium ion generated from **11** and isobutyric anhydride is expected to be the reactive intermediate which is responsible for the asymmetric acylation. The most stable conformer **A** of the acylpyridinium ion was generated by a molecular modeling search (AMBER* force field with the GB/SA solvation model for chloroform using MacroModel V 9.0 (50,000 steps MCMM)) and shown in Figure 2a and Figure 2b. Since the amide carbonyl groups at C(2) and C(5) seem to play the key role in the asymmetric desymmetrization of **5** to give (1*R*,2*S*)-**6** (Table 1 and Table 4), we assume that the amide carbonyl group would serve as a hydrogen-bond acceptor and the non-reacting OH of **5** as a hydrogen-bond donor. A possible approach of substrate **5** to **A** is shown in Figure 2c and Figure 2d. In the case where a hydrogen bond between the amide carbonyl group and an axial-OH at the (*S*)-chiral center of **5** is formed, an equatorial-OH at the (*R*)-chiral center locates in the close proximity to the reactive carbonyl group without any unfavorable steric interaction, resulting in the selective acylation of the hydroxy group at the (*R*)-chiral center to give (1*R*,2*S*)-**6**. On the other hand, there may be other possible modes of the approach of **5** to **A**. They involve hydrogen-bonding interaction between the amide carbonyl group of **A** and (1) an equatorial-OH at the (*S*)-chiral center of **5**, (2) an axial-OH at the (*R*)-chiral center of **5**, or (3) an equatorial-OH at the (*R*)-chiral center of **5**. The first one would give (1*R*,2*S*)-**6**, while the latter two would give (1*S*,2*R*)-**6**. In these cases, however, unfavorable steric interaction is expected based on our molecular modeling study. It is also anticipated that an axial-OH may be the better hydrogen-bond donor than an equatorial-OH, according to the reported higher acidity of the axial hydroxy groups of cyclohexane derivatives [39]. An alternative model for the transition-state assembly is shown in Figure 3 where the amide NH group of **A** serves as a hydrogen-bond donor and the non-reacting OH of substrate **5** as a hydrogen-bond acceptor. This model could explain the difference between effective catalyst **11** and ineffective catalysts **15** and **16** (Table 4, entry 1 vs entries 2 and 3). However, the calculated distance between the amide NH group and the reactive amide carbonyl group of **A** seems too long (8.10 Å) for the accommodation of the 1,2-diol substructure (calculated distance between two oxygen atoms of the hydroxy groups: 2.66 Å). It is also difficult to find the reasons for the preferable acylation of the hydroxy group at the (*R*)-chiral center of **5** from this model. We prefer the model shown in Figure 2, however, the model in Figure 3 cannot be eliminated.

## Conclusion

We have developed an organocatalytic method for the acylative asymmetric desymmetrization of *meso*-diols. Highly enantioselective desymmetrization of *meso*-1,2-cyclohexanediol and *meso*-2,3-butanediol (matched substrates) was achieved while low to moderate enantioselectivity was observed in the asymmetric desymmetrization of *meso*-1,3-cyclohexanediol, *meso*-hydrobenzoin, and *meso*-1,2-cyclopentanediol (mismatched substrates). Organocatalytic enantioselective acylation of the matched substrates was proposed to proceed via hydrogen-bonding interaction between the catalyst and the substrate.

## Supporting Information

File 1 Experimental details and characterization data of new compounds, copies of ^1^H NMR and ^13^C NMR.
