# Supplementary material for: Asymmetric desymmetrization of meso-diols by C2-symmetric chiral 4-pyrrolidinopyridines
Source: Beilstein J Org Chem. 2012 Oct 17;8:1778–87. doi: 10.3762/bjoc.8.203 (PMC3511012; doi:10.3762/bjoc.8.203)
Supplement: File 1 — Experimental details and characterization data of new compounds, copies of 1H NMR and 13C NMR. [file Beilstein_J_Org_Chem-08-1778-s001.pdf]

Supporting Information

for

Asymmetric desymmetrization of *meso*-diols by C_2-symmetric chiral 4-pyrrolidinopyridines

Hartmut Schedel, Keizo Kan, Yoshihiro Ueda, Kenji Mishiro, Keisuke Yoshida, Takumi Furuta, and Takeo Kawabata*

Address: Institute for Chemical Research, Kyoto University, Uji, Kyoto 611-0011, Japan

Email: Takeo Kawabata - kawabata@scl.kyoto-u.ac.jp

* Corresponding author

Experimental details and characterization data of new compounds, copies of ^{1}H NMR and ^{13}C NMR

Content:

General S2
List of abbreviation S2
General procedure for the preparation of C_2-symmetric catalysts S3
(2*S*,5*S*)-2,5-Bis[(2*S*)-3-(1*H*-indol-3-yl)-1-(methyloxy)-1-oxopropan-2-ylamino]-carbonyl]-1-(pyridin-4-yl)pyrrolidine (**8a**) S3
(2*S*,5*S*)-2,5-Bis[(2*R*)-3-(1*H*-indol-3-yl)-1-(methyloxy)-1-oxopropan-2-ylamino]carbonyl]-1-(pyridin-4-yl)pyrrolidine (**8b**) S3
(2*S*,5*S*)-2,5-Bis[tryptamino]carbonyl]-1-(pyridin-4-yl)pyrrolidine (**9**) S4
(2*S*,5*S*)-2,5-Bis[(2*S*)-3-(1-(methyloxy)-1-oxoethan-2-ylamino)carbonyl]-1-(pyridin-4-yl)pyrrolidine (**10**) S4
(2*S*,5*S*)-2,5-Bis[hexylamino]carbonyl]-1-(pyridin-4-yl)pyrrolidine (**11**) S5
(2*S*,5*S*)-2,5-Bis[(2*S*)-3-(2-naphthylmethyl)-1-(methyloxy)-1-oxopropan-2-ylamino]-carbonyl]-1-(pyridin-4-yl)pyrrolidine (**12a**) S5
(2*S*,5*S*)-2,5-Bis[(2*R*)-3-(2-naphthylmethyl)-1-(methyloxy)-1-oxopropan-2-ylamino]carbonyl]-1-(pyridin-4-yl)pyrrolidine (**12b**) S6
(2*S*,5*S*)-2,5-Bis[(2*S*)-3-(isopropyl)-1-(methyloxy)-1-oxopropan-2-ylamino]-carbonyl]-1-(pyridin-4-yl)pyrrolidine (**13**) S6
(2*S*,5*S*)-2,5-Bis[(2*S*)-3-(isobutyl)-1-(methyloxy)-1-oxopropan-2-ylamino]-carbonyl]-1-(pyridin-4-yl)pyrrolidine (**14**) S7

(2*S*,5*S*)-2,5-Bis[*N*-methyl-*N*-hexylamino]carbonyl]-1-(pyridin-4-yl)pyrrolidine (**15**)............. S7
(2*S*,5*S*)-Dihexyl 1-(pyridin-4-yl)pyrrolidine-2,5-dicarboxylate (**16**).. S8
4-Pyridinyl-L-proline 1'-hexyloxyamide (**17**).. S8
Typical procedure for asymmetric desymmetrization... S9
1,3-Cyclohexanediol isobutyrate (**20**).. S9
Acylative kinetic resolution of racemic-**6** with catalyst **12b** S9
Determination of the absolute configuration of 2,3-butanediol isobutyrate (**23a**)..................... S9
2,3-Butanediol diisobutyrate (**24a**) S10
Conformation search of cat **11** and *meso*-1,2-cyclohexanediol S11
References... S11
^{1}H and ^{13}C NMR spectra... S12

Some of the results shown are already mentioned in the patent, JP2005132746.

General

NMR spectra were obtained with a Varian Gemini 200 (^{1}H NMR: 200 MHz, ^{13}C NMR: 50 MHz) or JEOL JMN 400 spectrometer (^{1}H NMR: 400 MHz, ^{13}C NMR: 100 MHz), chemical shifts being given in ppm units (tetramethylsilane or the solvent residual signal for CD_3OD as internal standard, indicating 0 and 3.30, respectively). IR spectra were recorded with a JASCO FT/IR-300 spectrometer. Specific rotation was measured with a Horiba SEPA-200 automatic digital polarimeter. MS spectra were recorded with a JEOL JMS-DX300 mass spectrometer or JEOL JMS–700 mass spectrometer. TLC analysis and preparative TLC were performed on commercial glass plates bearing a 0.25 mm layer and 0.5 mm layer of Merck Kiesel-gel 60 F_{254}, respectively. Silica gel chromatography was carried with Wakogel C-200, Fuji Silysia BW-1277H, or Nacalai Tesque Silica gel 60 (150–325 mesh). Dry solvents (THF, ether, hexane, dichloromethane, and toluene; <50 ppm water contents) were purchased from Kanto Chemical CO., Inc. and used without further treatment.

List of abbreviations

AcOEt	ethyl acetate
DCM	dichloromethane
DMAP	4-dimethylaminopyridine
DMF	dimethylformamide
EDCI	1-ethyl-3-(3-dimethylaminopropyl)carbodiimide hydrochloride
HOBt	1-hydroxybenzotriazole
NMM	*N*-methylmorpholine
THF	tetrahydrofuran

General procedure for the preparation of C_2-symmetric catalysts

To a solution of (2*S*,5*S*)-1-(pyridin-4-yl)pyrrolidine-2,5-dicarboxylic acid [1] (130 mg, 0.55 mmol) and the corresponding amino acid or amino alcohol (1.6 mmol, 2.9 equiv) in DMF or DCM (10 mL) were added NMM (148 μl, 1.6 mmol, 2.9 equiv), EDCI (312 mg, 1.6 mmol, 2.9 equiv) and HOBt (222 mg, 1,6 mmol, 2.9 equiv). After stirring for 24 h, the mixture was evaporated and diluted with EtOAc (200 mL). The organic layer was washed with sat. aq. $NaHCO_3$, brine, dried over Na_2SO_4, filtered and evaporated in vacuo. The residue was purified by SiO_2 column chromatography or preparative TLC on silica gel, or recrystallization to give the corresponding C_2-symmetric catalyst.

Preparation of (2*S*,5*S*)-1-(pyridin-4-yl)pyrrolidine-2,5-dicarboxylic acid was previously reported [1].

(2*S*,5*S*)-2,5-Bis[(2*S*)-3-(1*H*-indol-3-yl)-1-(methyloxy)-1-oxopropan-2-ylamino]carbonyl]-1-(pyridin-4-yl)pyrrolidine (8a)

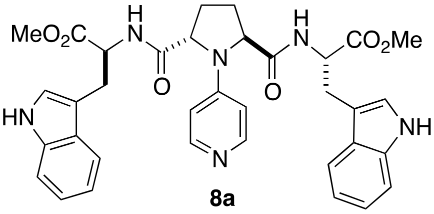

8a

According to the general procedure, **8a** was prepared with (*S*)-tryptophane methyl ester hydrochloride in DMF and purified by SiO_2 column chromatography (AcOEt/MeOH = 5/1) as a white powder in 46% yield.

M.p. 223–225 °C. $[\alpha]_D^{20}$ = –26 (*c* 0.5, DMSO). ^{1}H NMR (200 MHz, DMSO) δ 1.65–1.85 (m, 2H), 2.20 (br s, 2H), 3.05 (dd, *J* = 10.6, 14.6 Hz, 1H), 3.24 (dd, *J* = 4.2, 14.6 Hz, 1H), 4.22 (d, *J* = 7.8 Hz, 1H), 4.20–4.60 (m, 1H), 5.78 (d, *J* = 6.0 Hz, 1H), 6.90-7.16 (m, 2H), 7.21 (s, 1H), 7.38 (d, *J* = 8.0 Hz, H), 7.55 (d, *J* = 7.2 Hz, H), 7.74 (d, *J* = 5.0 Hz, 1H), 8.68 (d, *J* = 8.4 Hz, 1H). ^{13}C NMR (50 MHz, DMSO) δ 24.3, 26.6, 49.6, 50.4, 58.6, 104.9, 107.3, 109.0, 115.5, 116.0, 118.5, 121.3, 124.5, 133.7, 146.2, 147.5, 169.4, 169.7. IR (KBr) 3255, 1755, 1735, 1655, 1600 cm^{-1}. MS (FAB) *m/z* (rel intensity) 659 (MNa^+), 637 (MH^+). HRMS (FAB) calcd for $C_{35}H_{37}N_6O_6$ (MH): 637.2775, found, 637.2764. Anal calcd for $C_{35}H_{36}N_6O_6$: C, 66.03; H, 5.70; N, 13.20. Found C, 65.91; H, 5.79; N, 13.00.

(2*S*,5*S*)-2,5-Bis[(2*R*)-3-(1*H*-indol-3-yl)-1-(methyloxy)-1-oxopropan-2-ylamino]carbonyl]-1-(pyridin-4-yl)pyrrolidine (8b)

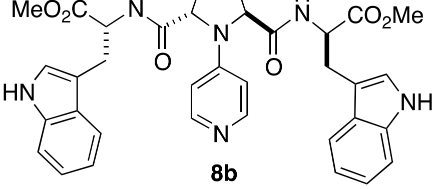

8b

According to the general procedure, **8b** was prepared with (*R*)-tryptophane methyl ester hydrochloride in DMF and purified by SiO_2 column chromatography (AcOEt/MeOH = 5/1)

in 34% yield as a crystalline powder.
M.p. 176–178 °C. $[\alpha]_D^{20}$ = –58 (c 0.5, DMSO). ^{1}H NMR (200 MHz, CD_3OD) δ 1.40–1.70 (m, 2H), 2.03 (br s, 2H), 3.12 (dd, *J* = 9.4, 14.6 Hz, 2H), 3.20–3.40 (m, 1H), 3.70 (s, 6H), 4.29 (d, *J* = 7.8 Hz, 2H), 4.75 (dd, *J* = 4.8, 9.2, 2H), 6.28 (d, *J* = 6.8 Hz, 2H), 6.80-7.20 (m, 8H), 7.33 (d, *J* = 8.0 Hz, 2H), 7.45 (d, *J* = 7.4 Hz, 2H), 7.93 (d, *J* = 6.8 Hz, 2H). ^{13}C NMR (50 MHz, CD_3OD) δ 26.2, 28.4, 51.0, 52.5, 61.6, 107.9, 108.7, 110.5, 117.1, 118.0, 120.6, 122.4, 126.6, 136.1, 144.9, 151.7, 171.7, 172.0. IR (KBr) 3380, 3260, 1740, 1665, 1600 cm^{-1}. MS (FAB) *m/z* (rel intensity) 637 (MH^+). HRMS (FAB) calcd for $C_{35}H_{37}O_6N_4$ (MH): 637.2775, found, 637.2748.

(2*S*,5*S*)-2,5-Bis[tryptamino]carbonyl]-1-(pyridin-4-yl)pyrrolidine (9)

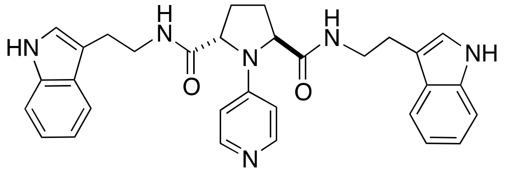

9

According to the general procedure, **9** was prepared with tryptamin hydrochloride in DMF and purified by SiO_2 column chromatography (AcOEt/MeOH = 5/1) in 40% yield as a crystalline powder.
M.p. 140–145 °C. $[\alpha]_D^{20}$ = –36 (c 0.5 MeOH). ^{1}H NMR (200 MHz, CD_3OD) δ 1.70–1.90 (m, 2H), 2.10–2.40 (m, 2H), 2.91 (t, *J* = 7.6 Hz, 4H), 3.49 (dd, *J* = 7.0 Hz, 4H), 4.31 (d, *J* = 7.6 Hz, 2H), 6.16 (d, *J* = 6.6 Hz, 2H), 6.90–7.20 (m, 6H), 7.30–7.40 (m, 2H), 7.42–7.60 (m, 2H), 7.85 (d, 6.6, 2H). ^{13}C NMR (50 MHz, CD_3OD) δ 24.1, 28.7, 39.2, 61.8, 107.6, 110.4, 111.0, 117.4, 117.8, 120.5, 121.7, 126.9, 136.2, 147.3, 150.7, 172.4. IR (KBr) 3240, 1660, 1600, 1518 cm^{-1}. MS (FAB) *m/z* (rel intensity) 521 (MH^+). HRMS (FAB) calcd for $C_{31}H_{32}O_2N_6$ (MH): 521.2665, found, 521.2657.

(2*S*,5*S*)-2,5-Bis[(2*S*)-3-(1-(methyloxy)-1-oxoethan-2-ylamino)carbonyl]-1-(pyridin-4-yl) pyrrolidine (10)

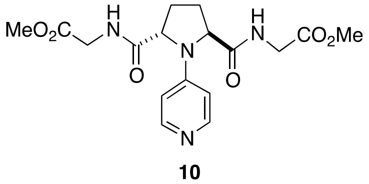

10

According to the general procedure, **10** was prepared with glycine methyl ester hydrochloride in 16% yield as a white powder.
M.p. 222–225 °C. $[\alpha]_D^{20}$ = –76 (c 0.25, CH_3OH). ^{1}H NMR (200 MHz, CD_3OD) δ 2.10–2.30 (m, 2H), 2.42–2.33 (m, 2H), 3.73 (s, 6H), 3.96 (d, *J* = 6.2 Hz, 4H), 4.59 (d, *J* = 8.2 Hz, 2H), 6.56 (d, *J* = 6.4 Hz, 2H), 8.14 (d, *J* = 6.4 Hz, 2H). ^{13}C NMR (50 MHz, CD_3OD) δ 28.7, 39.9, 50.7, 61.7, 108.1, 147.2, 150.8, 169.5, 173.4. IR (KBr) 3245, 1742, 1642, 1600, 1542, 1519 cm^{-1}. MS (FAB) *m/z* (rel intensity) 401 (MNa^+, 12), 379 (MH^+, 100), 262 (30). HRMS (FAB) calcd for $C_{17}H_{23}O_6N_4$ (MH): 379.1618, found, 379.1598.

(2*S*,5*S*)-2,5-Bis[hexylamino]carbonyl]-1-(pyridin-4-yl)pyrrolidine (11)

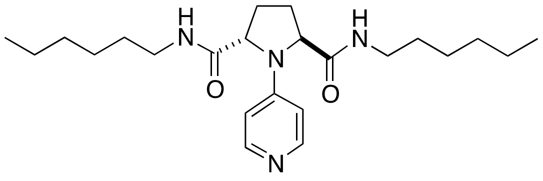

11

According to the general procedure, **11** was prepared with *n*-hexylamine in DCM, purified by repeated preparative TLC (SiO_2, EtOAc/MeOH = 3 : 1 and $CHCl_3$/MeOH = 5 : 1) and recrystallization from EtOAc-CH_2Cl_2 to afford **11** in 54% yield as a colorless powder.
M.p. 222–223 °C. $[\alpha]_D^{20}$ = –86 (c 0.5, $CHCl_3$). ^{1}H NMR (200 MHz, $CDCl_3$) δ 0.85 (t, *J* = 6.8 Hz, 6H), 1.10–1.35 (m, 12H), 1.35–1.50 (m, 4H), 2.10–2.30 (m, 2H), 2.30–2.60 (m, 2H), 3.10–3.40 (m, 4H), 4.42 (d, *J* = 7.6 Hz, 2H), 6.30–6.60 (m, 4H), 8.13 (dd, *J* = 1.4, 5.0 Hz, 2H). ^{13}C NMR (50 MHz, $CDCl_3$) δ 14.1, 22.7, 26.7, 29.6, 30.1, 31.6, 39.7, 62.4, 108.3, 149.0, 150.5, 172.3. IR (KBr) 3288, 2928, 1651, 1596, 1552, 1510 cm^{-1}. MS (FAB) *m/z* (rel intensity) 402 (M, 3), 274 (100), 145 (60). HRMS (FAB) calcd for $C_{23}H_{38}O_2N_4$ (M^+) 402.2995, found, 402.2974. Anal calcd for $C_{23}H_{38}N_2O_4$: C, 68.62; H, 9.51; N, 13.92. Found C, 68.35; H, 9.40; N, 13.93.

(2*S*,5*S*)-2,5-Bis[(2*S*)-3-(2-naphthylmethyl)-1-(methyloxy)-1-oxopropan-2-ylamino]carbonyl]-1-(pyridin-4-yl)pyrrolidine (12a)

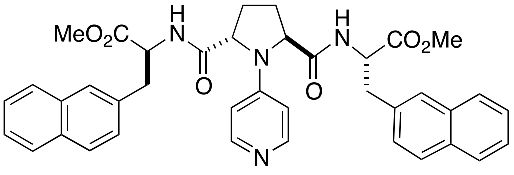

12a

According to the general procedure, **12a** was prepared with (*S*)-naphtylalanine methyl ester hydrochloride in DMF in 41% yield as a white powder.
M.p. 225–228 °C. $[\alpha]_D^{20}$ = +39 (c 0.25, MeOH). ^{1}H NMR (200 MHz, DMSO) δ 1.50–1.65 (m, 2H), 2.09 (br s, 2H), 3.05 (t, *J* = 13.0 Hz, 2H), 3.20–3.50 (m, 2H), 3,63 (s, 6H), 4.10 (d, *J* = 7.2 Hz, 2H), 4.50–4.65 (m, 2H), 5.53 (d, *J* = 6.2 Hz, 2H), 7.10 (d, *J* = 5.8 Hz, 2H), 7.38-7.60 (m, 6H), 7.70–8.00 (m, 8H), 8.66 (d, *J* = 8.4 Hz, 2H). ^{13}C NMR (50 MHz, DMSO) δ 29.6, 37.1, 52.9, 53.7, 61.9, 108.0, 126.34, 126.8, 128.1, 128.3, 128.4, 132.6, 133.6, 135.9, 149.2, 150.4, 172.4, 172.5. IR (KBr) 3255, 1750, 1650, 1600 cm^{-1}. MS (FAB) *m/z* (rel intensity) 659 (MH^+). HRMS (FAB) calcd for $C_{39}H_{39}O_6N_4$ (MH): 659.2869, found, 659.2859.

(2*S*,5*S*)-2,5-Bis[(2*R*)-3-(2-naphthylmethyl)-1-(methyloxy)-1-oxopropan-2-ylamino]carbonyl]-1-(pyridin-4-yl)pyrrolidine (12b)

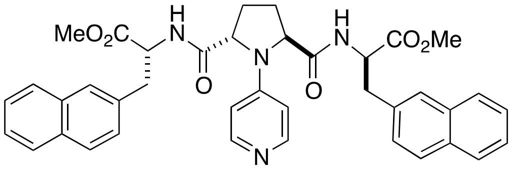

12b

According to the general procedure, **12b** was prepared with (*R*)-naphtylalanine methyl ester hydrochloride in DMF and purified by SiO_2 column chromatography (SiO_2, AcOEt /MeOH = 4/1) in 53% yield as an amorphous powder.
M.p. 191–193 °C. $[\alpha]_D^{20}$ = –90 (c 0.5, MeOH). ^{1}H NMR (200 MHz, CD_3OD) δ 1.20–1.50 (m, 2 H), 1.87 (br s, 2 H), 3.05 (dd, *J* = 10.8, 13.9 Hz, 2 H), 3.41 (dd, *J* = 4.4, 13.9 Hz, 2H), 3.73 (s, 6H), 4.26 (d, *J* = 7.6 Hz, 2H), 4.83 (dd, *J* = 4.6, 10.8 Hz, 2H), 6.18 (d, *J* = 6.6 Hz, 2H), 7.25 (dd, *J* = 1.6, 8.4 Hz, 2H), 7.40–7.60 (m, 6H), 7.60–7.90 (m, 8H). ^{13}C NMR (100 MHz, CD_3OD) δ 28.4, 36.2, 51.1, 52.6, 61.4, 107.7, 124.9, 125.3, 126.2, 126.6, 126.7, 126.8, 127.2, 131.89, 132.9, 133.8, 146.7, 150.7, 171.3, 172.4. IR (KBr) 3280, 1740, 1657, 1600 cm^{-1}. MS *m/z* (FAB) (rel intensity) 659 (MH^+). HRMS (FAB) calcd for $C_{39}H_{39}O_6N_4$ (MH): 659.2869, found, 659.2859.

(2*S*,5*S*)-2,5-Bis[(2*S*)-3-(isopropyl)-1-(methyloxy)-1-oxopropan-2-ylamino]-carbonyl]-1-(pyridin-4-yl)pyrrolidine (13)

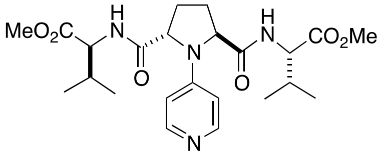

13

According to the general procedure, **13** was prepared with (*S*)-valine methyl ester hydrochloride in CH_2Cl_2 with preparative TLC (SiO_2, AcOEt /MeOH = 3/1) in 29% yield as a white powder.
M.p. 203–205 °C. $[\alpha]_D^{21}$ = –117 (c 0.5, MeOH). ^{1}H NMR (200 MHz, CD_3OD) δ 0.99–1.10 (m, 12H), 2.07–2.35 (m, 4H), 2.40–2.60 (m, 2H), 3.73 (s, 6H), 4.37 (d, *J* = 5.6 Hz, 2H), 4.66 (d, *J* = 7.6 Hz, 2H), 6.47 (d, *J* = 6.4 Hz, 2H), 8.09 (d, *J* = 6.4 Hz, 2H). ^{13}C NMR (50 MHz, CD_3OD) δ 16.7, 17.8, 28.7, 29.7, 50.6, 57.4, 61.2, 107.5, 147.3, 151.0, 171.3, 173.1. IR (KBr) 3255, 2880, 1740, 1655, 1600 cm^{-1}. MS (FAB) *m/z* (rel intensity) 462 (M, 7), 304 (55), 71 (100). HRMS (FAB) Calcd for $C_{23}H_{34}O_6N_4$ (M): 462.2478, found, 462.2490.

(2*S*,5*S*)-2,5-Bis[(2*S*)-3-(isobutyl)-1-(methyloxy)-1-oxopropan-2-ylamino]-carbonyl]-1-(pyridin-4-yl)pyrrolidine (14)

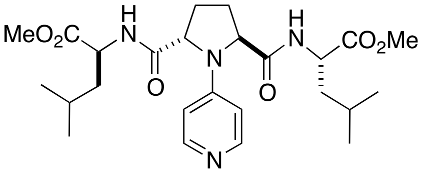

14

According to the general procedure, **14** was prepared with (*S*)-leucine methyl ester hydrochloride in CH_2Cl_2 with SiO_2 column chromatography ($CHCl_3$/MeOH = 6/1) and successive preparative TLC (SiO_2, $CHCl_3$ /MeOH = 6/1) in 71% yield as a white powder.
M.p. 187–189 °C. $[\alpha]_D^{20}$ = –133 (c 0.5, CH_3OH). ^{1}H NMR (200 MHz, CD_3OD) δ 0.93 (d, *J* = 5.8 Hz, 6H), 1.02 (d, *J* = 6.2 Hz, 6H), 1.50–1.90 (m, 4H), 2.10–2.30 (m, 2H), 2.35–2.60 (m, 2H), 3.70 (s, 6H), 4.20–4.60 (m, 4H), 6.46 (d, *J* = 5.5 Hz, 2H), 8.10 (d, *J* = 5.5 Hz, 2H). ^{13}C NMR (50 MHz, CD_3OD) δ 19.6, 21.6, 24.4, 28.6, 39.1, 50.2, 50.8, 61.4, 107.7, 147.1, 151.0, 172.2, 172.93. IR (KBr) 2880, 1745, 1660, 1600, 1545, 1518 cm^{-1}. MS (FAB) *m/z* (rel intensity) 490 (M, 4), 318 (100), 145 (40). HRMS (FAB) calcd for $C_{25}H_{38}O_6N_4$ (M): 490.2791, found, 490.2796.

(2*S*,5*S*)-2,5-Bis[*N*-methyl-*N*-hexylamino]carbonyl]-1-(pyridin-4-yl)pyrrolidine (15)

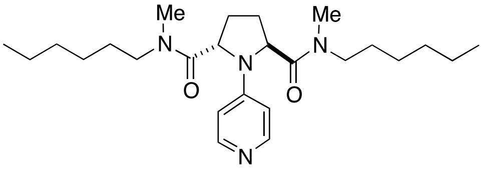

15

According to the general procedure, **15** was prepared with *N*-methyl hexylamine in CH_2Cl_2 without addition of NMM and purified by preparative TLC (SiO_2, $CHCl_3$/MeOH = 5/1) in 42% yield as a white solid.
Decomposition. $[\alpha]_D^{20}$ = –28 (c 0.5, $CHCl_3$). ^{1}H NMR (400 MHz, DMSO, 150 °C) δ 0.90 (t, *J* = 6.8 Hz, 6H), 1.20–1.40 (m, 12H), 1.45–1.65 (m, 4H), 1.80–1.95 (m, 2H), 2.40–3.15 (m, 2H), 3.04 (m, 6H), 3.38 (t, *J* = 6.8 Hz, 4H), 4.81 (d, *J* = 7.8 Hz, 2H), 6.12 (d, *J* = 6.2 Hz, 2H), 8.02 (d, *J* = 6.2 Hz, 2H). ^{13}C NMR (50 MHz, $CDCl_3$) δ 14.1, 22.7, 26.6, 26.8, 27.2, 28.5, 28.8, 29.2, 31.6, 33.9, 35.0, 48.4, 49.8, 59.2, 107.7, 149.6, 150.4, 171.1, 171.4. IR (KBr) 2931, 1650, 1603, 1513 cm^{-1}. MS (FAB) *m/z* (rel intensity) 430 (M, 4), 402 (2), 288 (92), 145 (100). HRMS (FAB) calcd for $C_{25}H_{42}O_2N_4$ (M): 430.3307, found, 430.3311.

(2*S*,5*S*)-Dihexyl 1-(pyridin-4-yl)pyrrolidine-2,5-dicarboxylate (16)

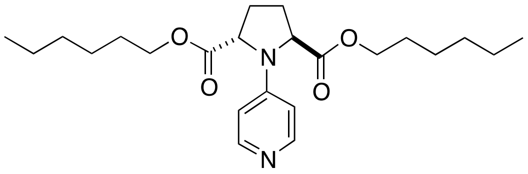

16

According to the general procedure, **16** was prepared with *n*-hexanol in CH_2Cl_2 without addition of NMM and purified by preparative TLC (SiO_2, Et_2O/MeOH = 10/1) in 38 % yield as a colorless oil.
$[\alpha]_D^{20}$ = –77 (c 0.25, $CHCl_3$). ^{1}H NMR (200 MHz, $CDCl_3$) δ 0.88 (t, *J* = 6.6 Hz, 6H), 1.00–1.43 (m, 12 H), 1.43–1.70 (m, 4H), 2.10–2.30 (m, 2H), 2.40–2.65 (m, 2H), 4.00–4.20 (m, 4H), 4.48 (d, *J* = 8.2 Hz, 2H), 6.33 (dd, *J* = 1.6, 4.8 Hz, 2H), 8.24 (dd, *J* = 1.4, 4.8 Hz, 2H). ^{13}C NMR (50 MHz, $CDCl_3$) δ 14.1, 22.6, 25.6, 28.7, 29.1, 31.4, 60.9, 65.7, 107.8, 150.0, 150.4, 172.7. IR (film) 2956, 2858, 1743, 1595, 1510 cm^{-1}. MS (FAB) *m/z* (rel intensity) 404 (M, 7), 275 (100), 191 (12), 145 (30). HRMS (FAB) calcd for $C_{23}H_{36}O_4N_2$ (M): 404.2675, found, 404.2673.

4-Pyridinyl-L-proline 1'-hexylamide (17)

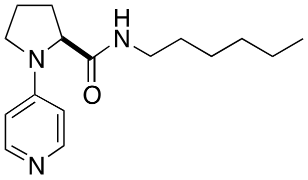

17

To a solution of *n*-hexylamine (87 µL, 0.66 mmol), *N*-(4-pyridyl)-L-proline hydrochloride (100 mg, 0.44 mmol) in DCM (10 mL) were added NMM (48 µl, 0.44 mmol), EDCI (125 mg, 0.66 mmol) and HOBt (89 mg, 0.66 mmol). After stirring for 24 h, the mixture was evaporated and diluted with EtOAc (200 mL). The organic layer was washed with sat. aq. $NaHCO_3$ (10 mL), brine (10 mL), dried over Na_2SO_4, filtered and evaporated in vacuo. The residue was purified by preparative TLC (SiO_2, $CHCl_3$/MeOH = 5/1) to give **17** (41 mg, 34%) as colorless needles.
M.p. 45 °C. $[\alpha]_D^{20}$ –110 (c 0.5, $CHCl_3$). ^{1}H NMR (200 MHz, $CDCl_3$) δ 0.85 (t, *J* = 6.4 Hz, 3 H), 1.10–1.35 (m, 6 H), 1.42 (t, *J* = 6.6 Hz, 2 H), 1.90–2.20 (m, 2 H), 2.20–2.50 (m, 2 H), 3.17-3.40 (m, 3 H), 3.50–3.80 (m, 1 H), 4.10 (t, *J* = 5.4 Hz, 1 H), 6.24 (br s, 1 H), 6.46 (d, *J* = 5.9 Hz, 2H), 8.27 (d, *J* = 5.9 Hz, 2H). ^{13}C NMR (50 MHz, NMR ($CDCl_3$) δ 14.1, 22.6, 24.0, 26.6, 29.7, 31.4, 31.5, 39.5, 48.9, 63.5, 108.2, 150.0, 152.1, 172.0. IR (KBr) 3299, 2928, 1650, 1602, 1551, 1518 cm^{-1}. MS (FAB) *m/z* (rel intensity) 275 (M^+, 42), 147 (100), 105 (40), 78 (38). HRMS (FAB) Calcd for $C_{16}H_{25}ON_3$ 275.1998, found 275.1999.

Typical procedure for asymmetric desymmetrization

To a solution of substrate (0.1 mmol), catalyst (5 mol %) and 2,4,6-collidine (0.14 mmol, 18.5 μL) in solvent (0.5 mL), isobutyric anhydride (0.13 mmol, 21.5 μL) was added. After the mixture was stirred, it was diluted with EtOAc and washed with 1 M aq. HCl, sat. aq. $NaHCO_3$ and brine. The organic layer was dried over Na_2SO_4, filtered and evaporated in vacuo. Yields of monoacylate, diacylate and recovered substrate were determined by ^{1}H NMR integration with dibenzyl ether as an internal standard.

Enantiomeric purities of 1,2- and 1,3-cyclohexanediol monoisobutyrate were determined by GLC analysis with beta-DEX 225 at 150 °C (Tables 1–5 in the manuscript).

Enantiomeric purities of *cis*-2,3-butanediol isobutyrate (**23a**) and hydrobenzoin isobutyrate (**23b**) were determined by GLC analysis with beta-DEX 225 at 90 °C and HPLC analysis with chiral stationary phase [column: CHIRALCEL OJ (4.6 mm x 25 cm), eluent: hexane–iPrOH (95:5), flow rate: 0.5 mL/min], respectively (Table 6 in the main manuscript).

1,3-Cyclohexanediol isobutyrate (20)

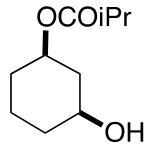

20

^{1}H-NMR (400 MHz, $CDCl_3$) δ: 4.65–4.54 (m, 1H), 3.61–3.50 (m, 1H), 3.28 (br s, 1H), 2.40 (sept, J = 7.1 Hz, 1H), 2.15–2.07 (m, 1H), 1.85–1.74 (m, 2H), 1.74–1.64 (m, 1H), 1.31–0.99 (m, 4H), 1.04 (d, J = 6.9 Hz, 6H). ^{13}C-NMR (100 MHz, $CDCl_3$) δ: 176.5, 70.6, 68.1, 40.4, 34.0, 33.9, 30.4, 19.9, 18.7. IR (neat) 3400, 2880, 2875, 2835, 1730, 1718 cm^{-1}; MS (EI) *m/z* (rel intensity) 186 (M^+, 0.15), 168 (5), 125 (10), 98 (80), 81 (100); HRMS (EI) calcd for $C_{10}H_{18}O_3$ (M^+), 186.1256, found 186.1248.

Acylative kinetic resolution of racemic-6 with catalyst 12b

To a solution of racemic-**6** (18.6 mg, 0.1 mmol), catalyst **12b** (3.3 mg, 5 mol%) and 2,4,6-collidine (13.2 μL, 0.1 mmol) in $CDCl_3$ (0.7 mL), isobutyric anhydride (11.6 μL, 0.07 mmol) was added at 20 °C. After the mixture was stirred at 20 °C for 23 h, conversion of **6** was determined by ^{1}H NMR integration with dibenzyl ether as an internal standard. Then, the mixture was diluted with EtOAc and washed with 1 M aq. HCl, sat. aq. $NaHCO_3$, and brine. The organic layer was dried over Na_2SO_4, filtered and evaporated in vacuo. Enantiomeric purity of (1*R*,2*S*)-**6** (67% ee) was determined by GLC analysis with beta-DEX 225 at 150 °C.

Determination of the absolute configuration of 2,3-butanediol isobutyrate (23a)

Asymmetric desymmetrization of 2,3-butanediol (**22a**) with benzoic anhydride in the presence of cat. **11**, according to the general procedure, afforded the monobenzoate (**S1**) in 50% ee in 39% yield (HPLC conditions: CHIRALPAK AD, *n*-hexane/2-propanol = 97:3, flow rate: 0.2 mL/min, t_R = 33 min, 37 min, Scheme I). The absolute configuration of **S1** was

determined to be (2*R*,3*S*) by comparison with the optical rotation of the literature data [2]. Monobenzoate (**S1**) was further converted to (2*R*,3*S*)-**S2** [$[\alpha]_D^{21}$ –13.8 (*c* 1.0, $CHCl_3$)]. On the other hand, 2,3-butanediol isobutyrate (**23a**) obtained in Table 5 was converted to the corresponding benzoate (**S2**), which was assigned to be (2*S*,3*R*) by comparison of the optical rotation of **S2** from Scheme I. Thus, the absolute configuration of **23a** was determined to be (2*R*,3*S*).

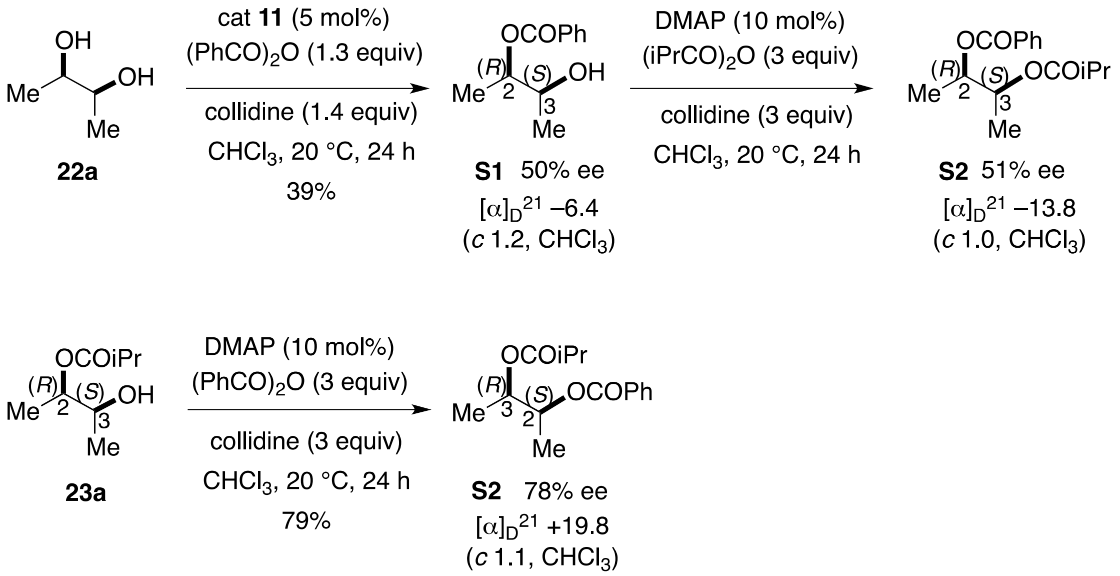

cis-2,3-Butanediol diisobutyrate (24a)

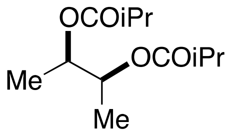

24a

^{1}H NMR (200 MHz, $CDCl_3$) δ 1.00-1.17 (m, 12H), 1.18 (d, *J* = 5.4 Hz, 6H), 2.40–2.62 (m, 2H), 4.90–5.10 (m, 2H). ^{13}C-NMR (100 MHz, $CDCl_3$) δ: 176.1, 70.8, 34.0, 18.9, 18.7, 14.9. IR (film) 2890, 2870, 2840, 1730 cm^{-1}. MS *m/z* (rel intensity) 230 (M, 0.03), 215 (2), 142 (70), 115 (72), 89 (68), 71 (100), 55 (58). HRMS calcd for $C_{12}H_{22}O_4$ 230.1518, found 230.1521. HRMS calcd for $C_{11}H_{19}O_4$ (M–CH_3) 215.1284, found 215.1287.

Conformation search of catalyst 11.

The most stable conformer of **11** was generated by a molecular modeling search with AMBER* force field with the GB/SA solvation model for chloroform using MacroModel V 9.0 (50,000 steps MCMM). Stereoview of the most stable conformation was shown below.

(a) side view

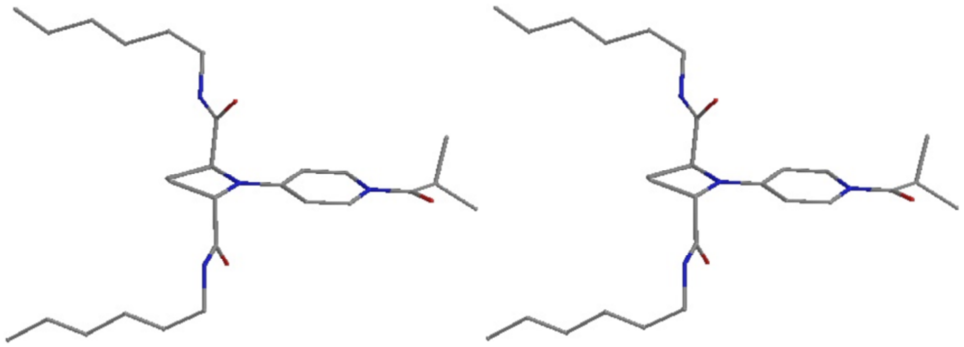

(b) front view

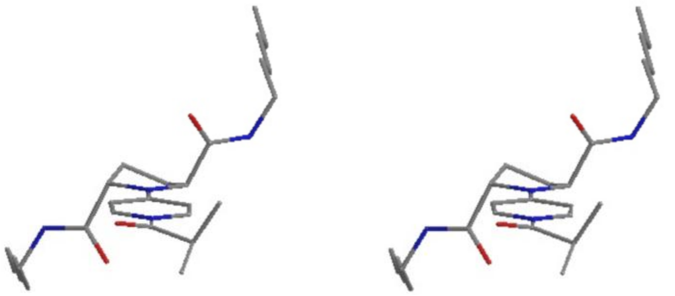

References

1. Kawabata, T.; Muramatsu, W.; Nishio, T.; Shibata, T.; Schedel, H. *J. Am. Chem. Soc.*, **2007**, *129*, 12890.
2. Mazet, C.; Köhler, V.; Pfaltz, A. *Angew. Chem. Int. Ed.* **2005**, *44*, 4888.

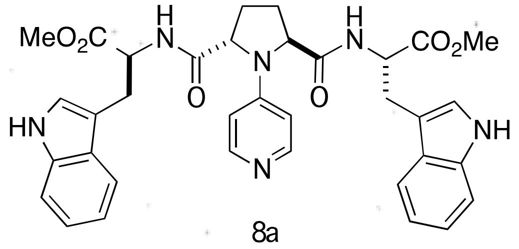
MeO2C
CO2Me
HN
NH
N
H
O
8a

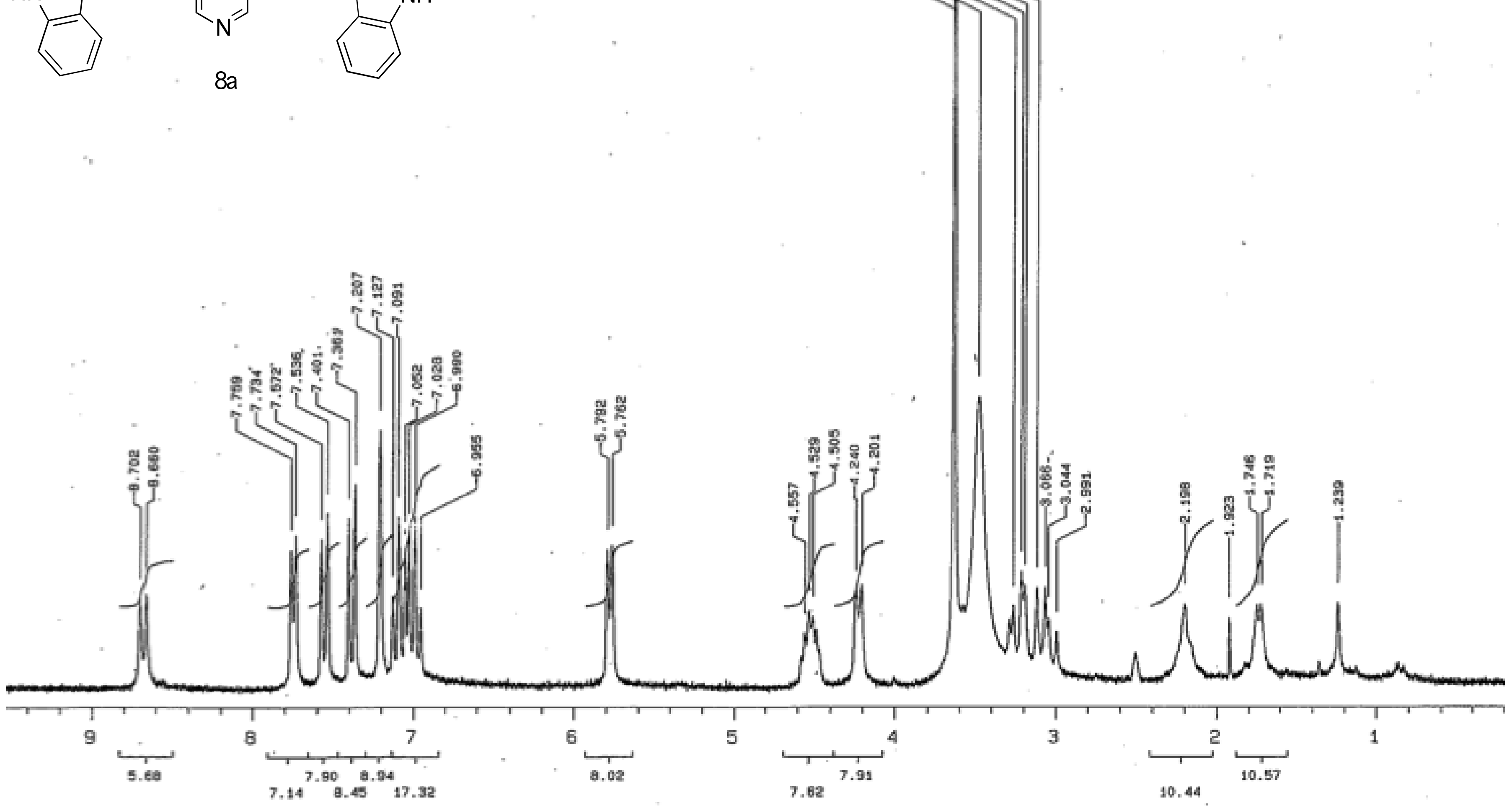
8.702
8.680
7.759
7.734
7.572
7.536
7.401
7.369
7.207
7.127
7.091
7.052
7.028
6.980
6.955
5.792
5.762
4.557
4.529
4.505
4.240
4.201
3.635
3.480
3.264
3.214
3.193
3.117
3.066
3.044
2.991
2.198
1.923
1.746
1.719
1.239
9
8
7
6
5
4
3
2
1
5.68
7.90
8.94
7.14
8.45
17.32
8.02
7.91
7.62
10.44
10.57

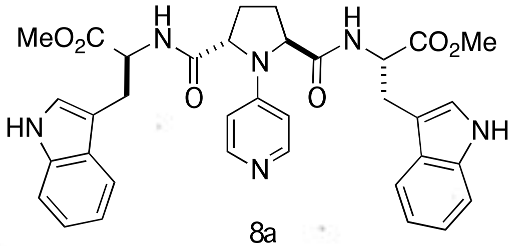
MeO$_2$C
H
N
O
N
O
H
N
CO$_2$Me
HN
N
NH

8a

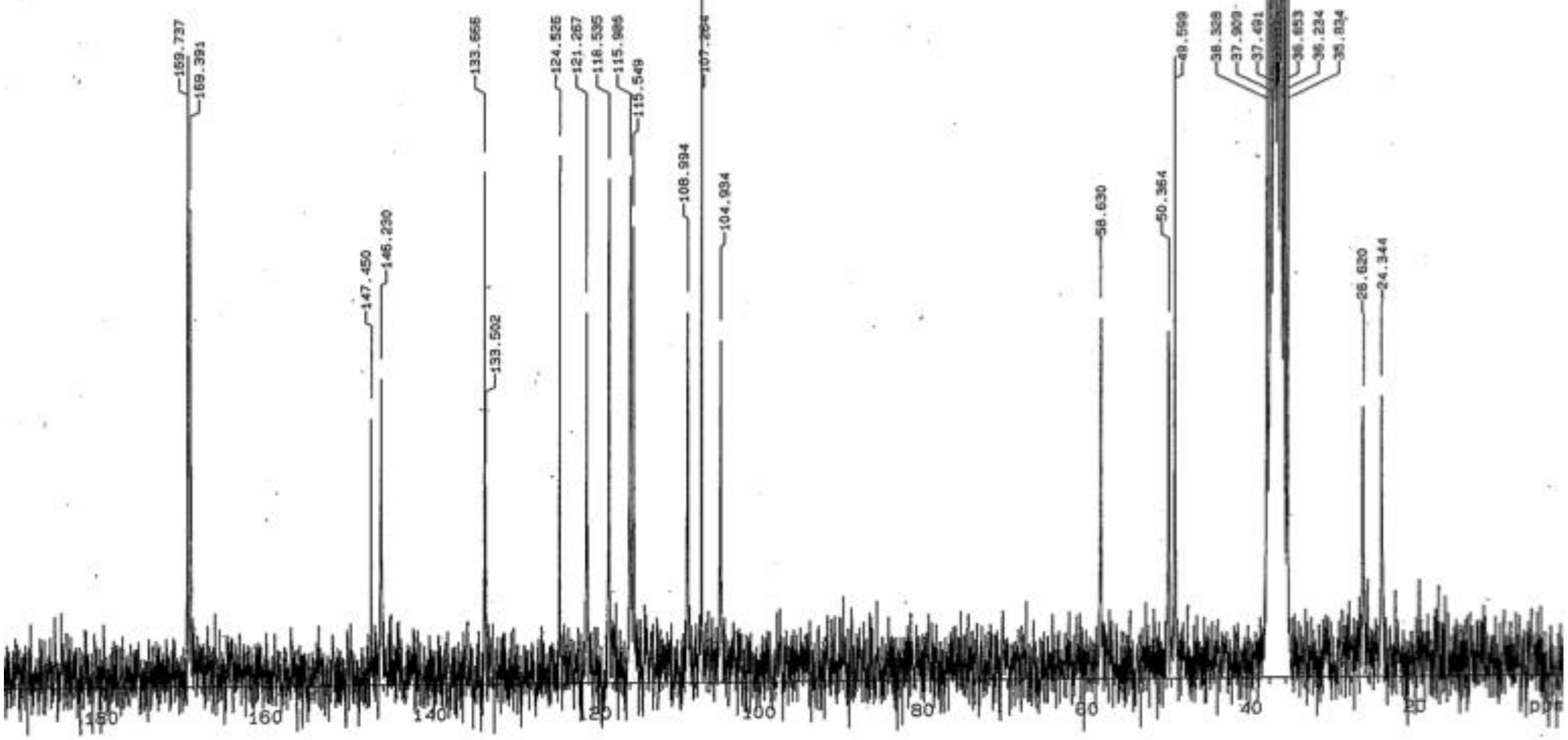
169.737
169.391
147.450
145.230
133.666
133.502
124.526
121.267
118.535
115.986
115.549
108.934
107.284
104.934
58.630
50.364
49.599
38.328
37.909
37.491
36.653
36.234
35.834
26.620
24.344
180
160
140
120
100
80
60
40
20
ppm

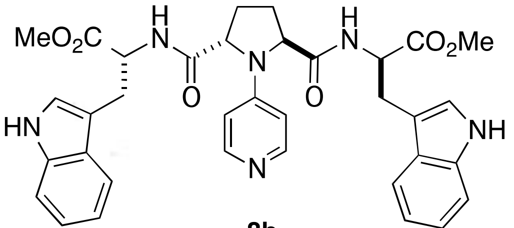
MeO2C
H
N
O
N
N
H
N
O
CO2Me
HN
NH

8b

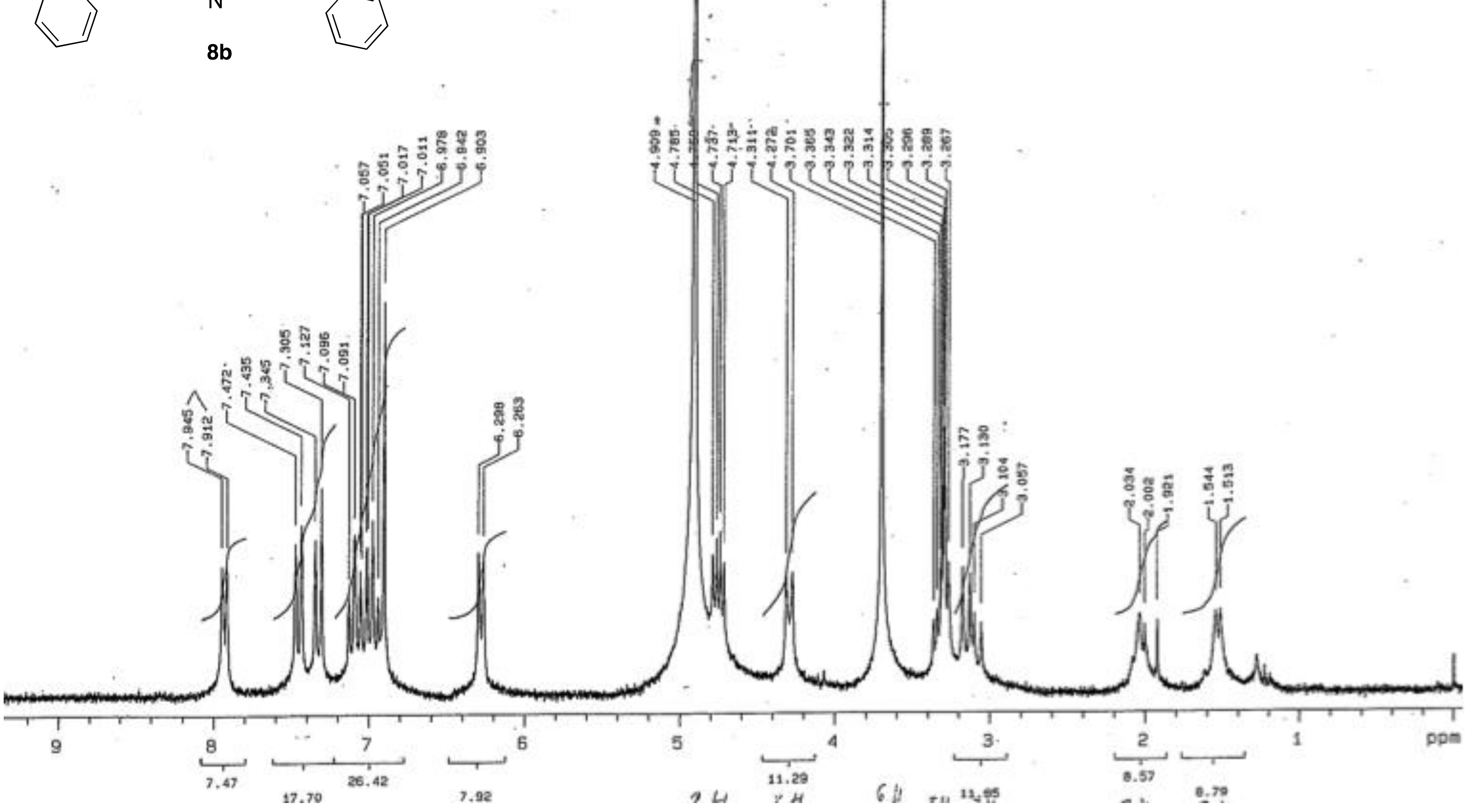
7.945
7.912
7.472
7.435
7.345
7.305
7.127
7.096
7.091
7.057
7.051
7.017
7.011
6.978
6.942
6.903
6.298
6.263
4.909
4.785
4.737
4.713
4.311
4.278
3.701
3.365
3.343
3.322
3.314
3.296
3.289
3.267
3.177
3.130
3.104
3.057
2.034
2.002
1.921
1.544
1.513
9
8
7
6
5
4
3
2
1
ppm
7.47
17.70
26.42
7.92
11.29
8.57
8.79

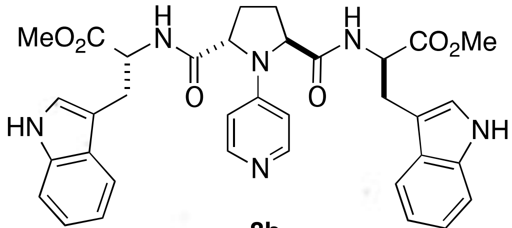
MeO2C
CO2Me
HN
NH
N
O
H

8b

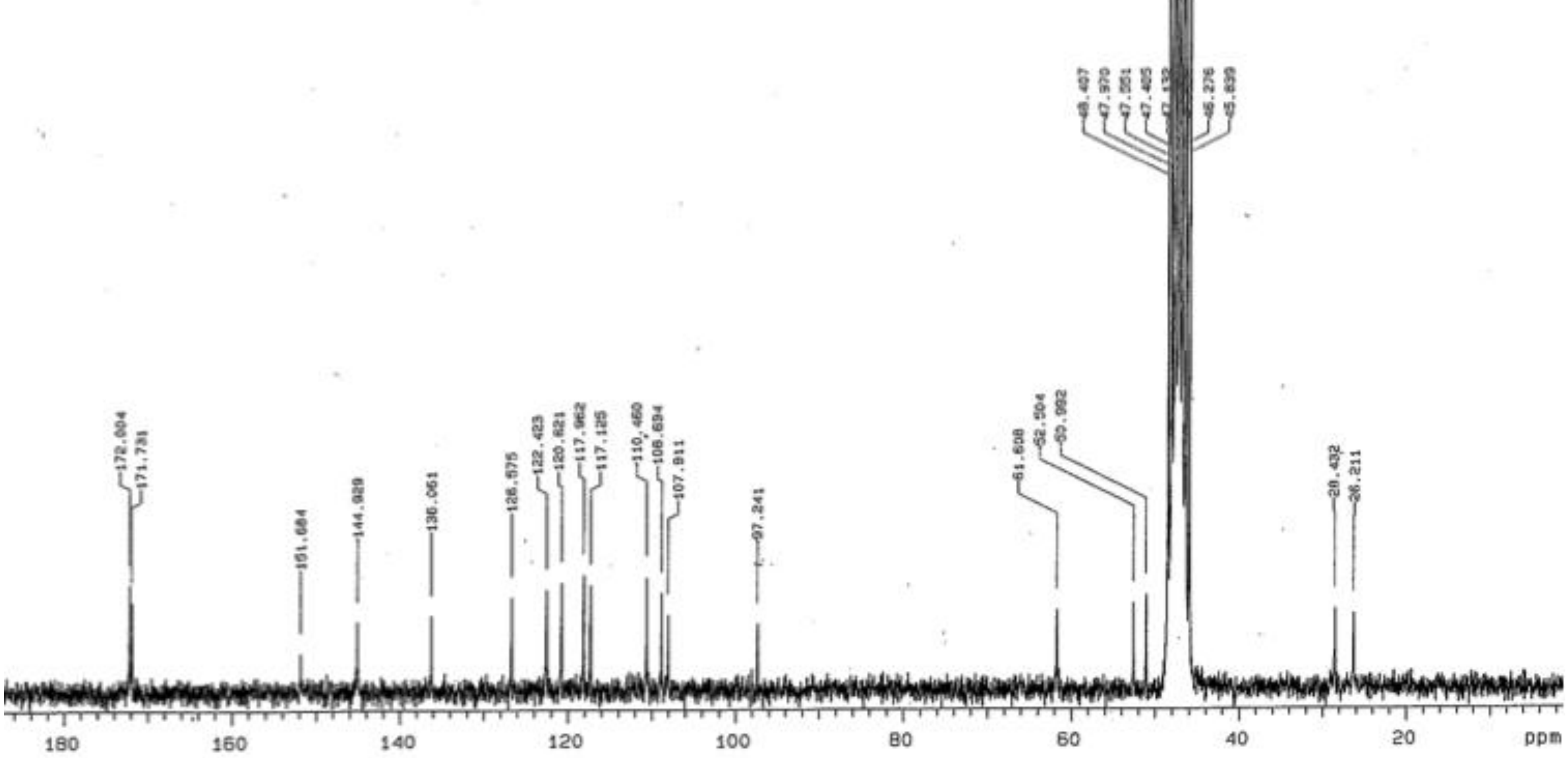
172.004
171.731
151.684
144.929
136.061
126.575
122.423
120.621
117.962
117.125
110.460
108.694
107.911
97.241
61.608
52.504
50.992
48.407
47.970
47.551
47.465
46.276
45.839
28.432
26.211
180
160
140
120
100
80
60
40
20
ppm

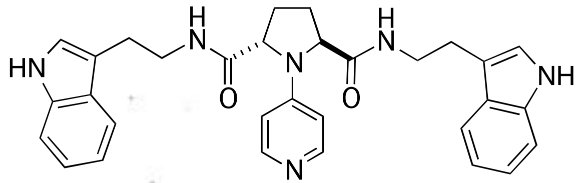
HN
H
N
O
N
NH

9

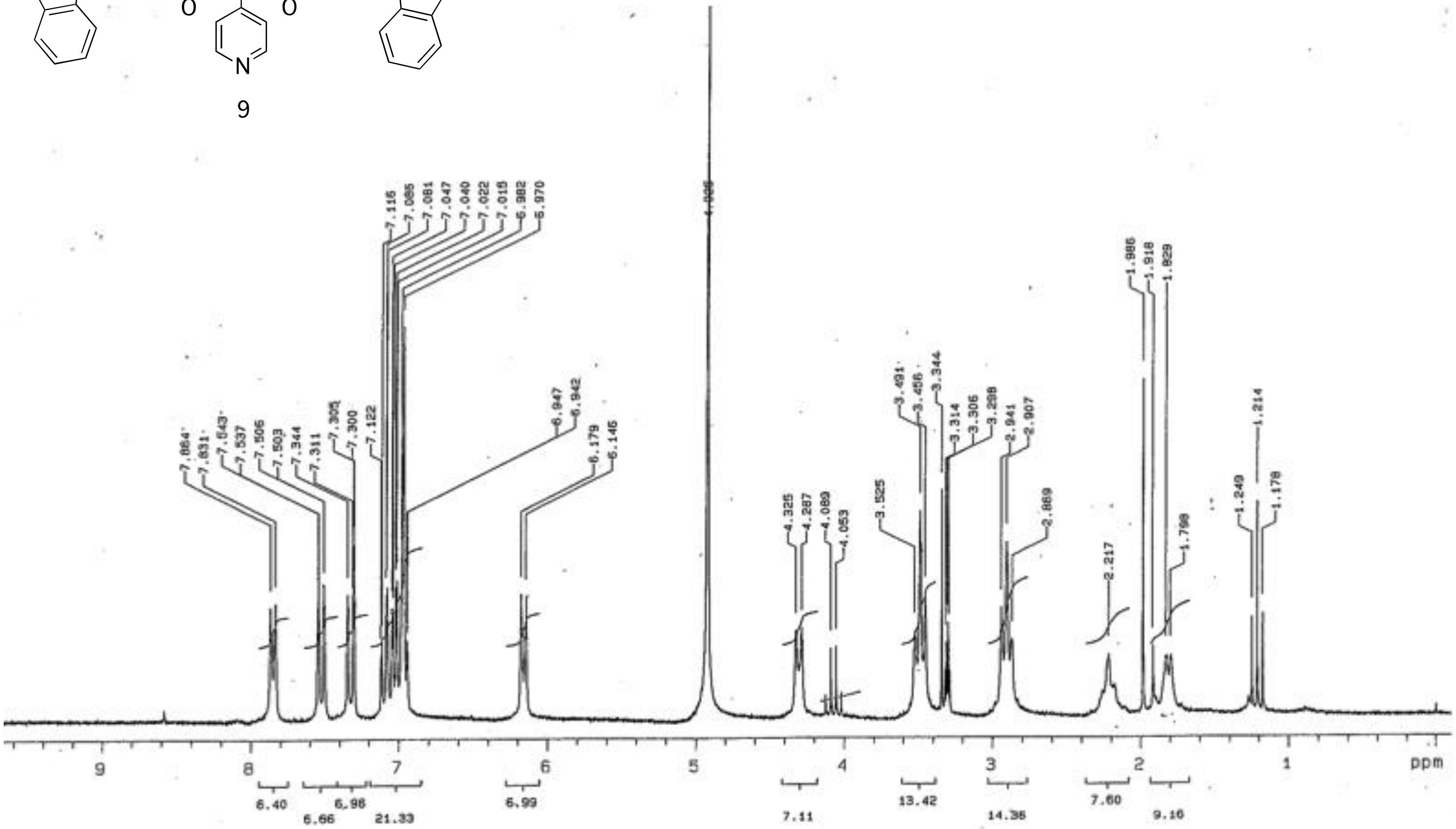
9
8
7
6
5
4
3
2
1
ppm
6.40
6.66
6.98
21.33
6.99
7.11
13.42
14.36
7.60
9.16

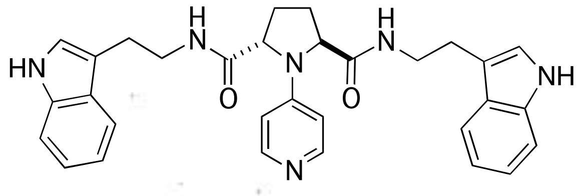

HN
H
N
O
N
N
H
N
O
NH

9

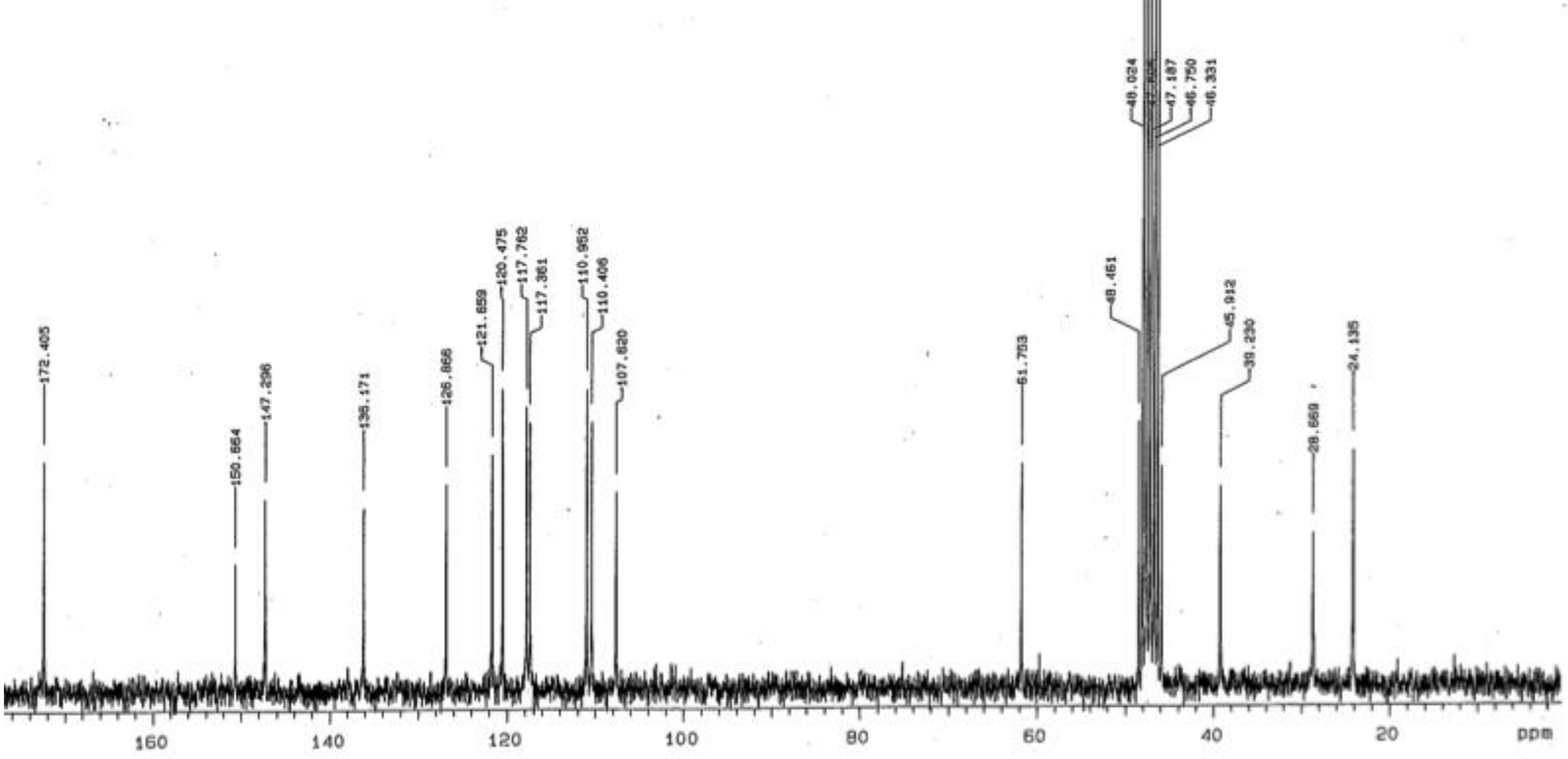

172.405
150.564
147.296
136.171
126.866
121.659
120.475
117.762
117.361
110.952
110.406
107.620
61.753
48.461
48.024
47.187
46.750
46.331
45.912
39.230
28.669
24.135
160
140
120
100
80
60
40
20
ppm

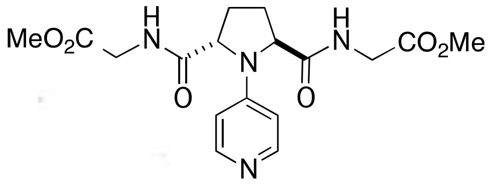
MeO2C
CO2Me
N
O
O
N

10

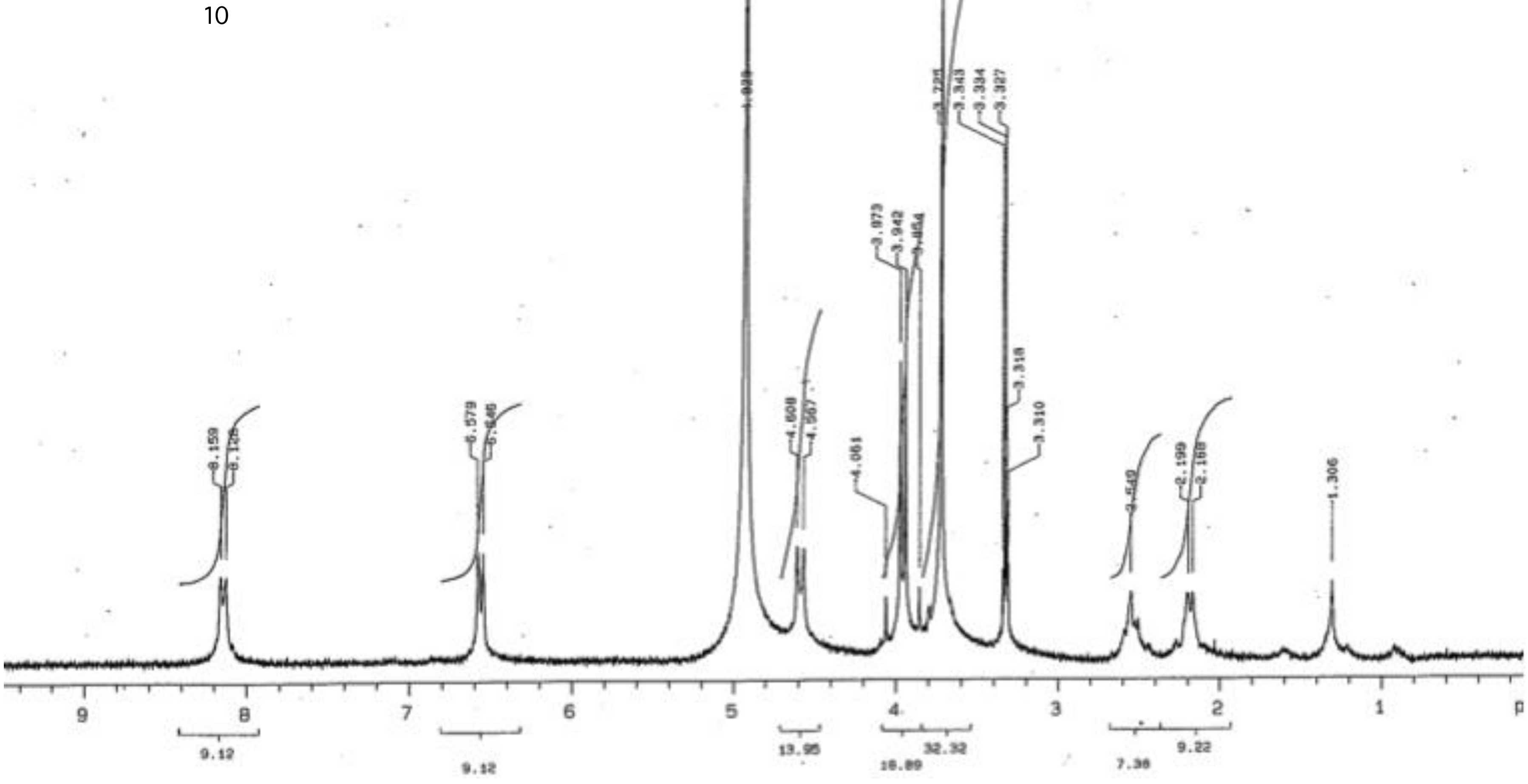
8.159
6.579
4.608
4.567
4.061
3.973
3.942
3.343
3.334
3.327
3.318
3.310
2.199
2.168
1.306
9
8
7
6
5
4
3
2
1
9.12
9.12
13.95
18.89
32.32
7.38
9.22

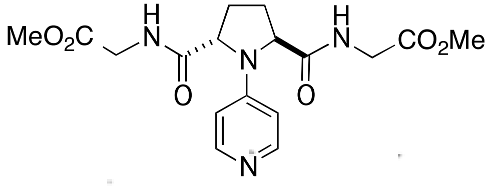

10

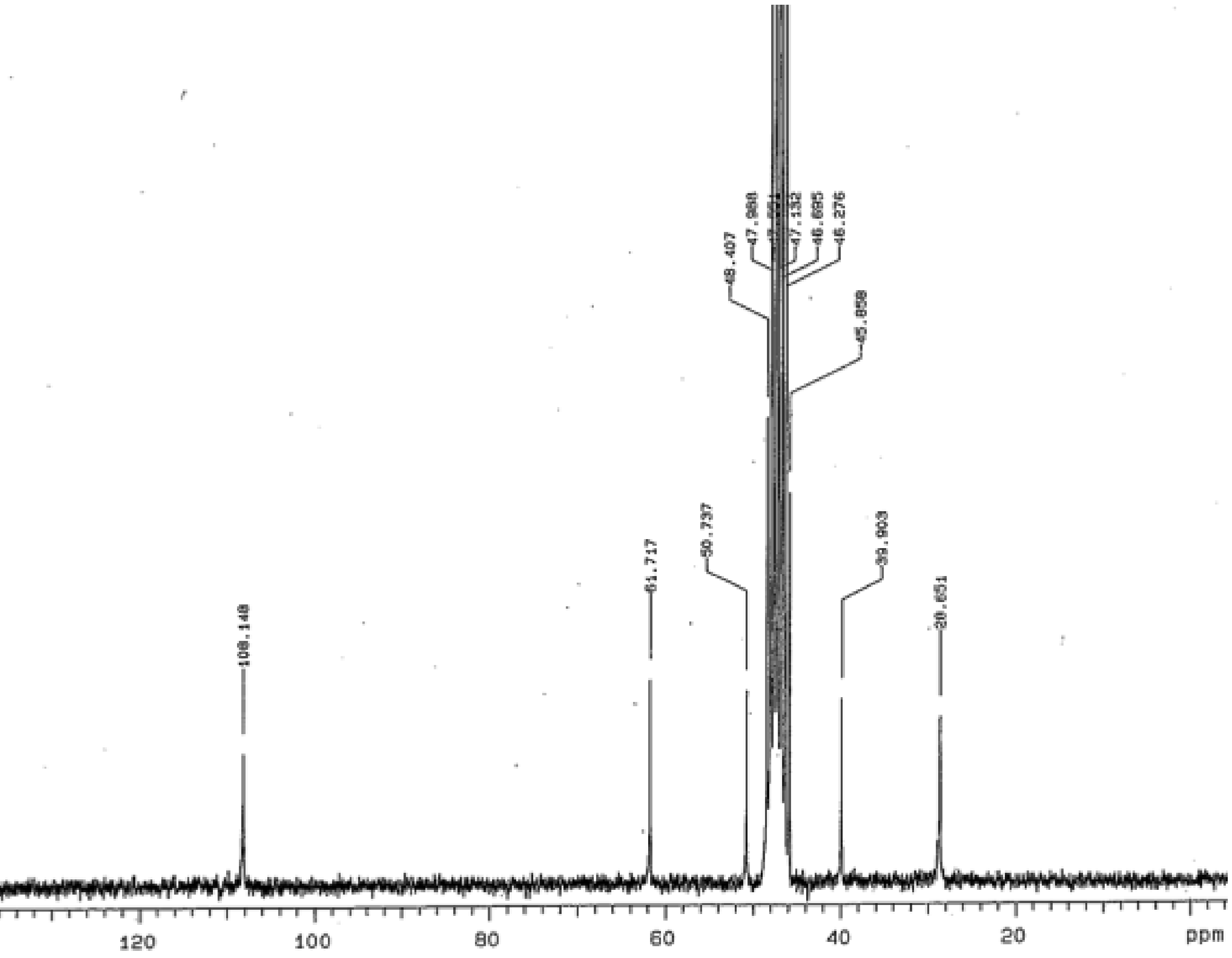

173.370
169.492
150.828
147.223
108.148
61.717
50.737
48.407
47.988
47.132
46.695
46.276
45.858
39.903
28.651
180
160
140
120
100
80
60
40
20
ppm

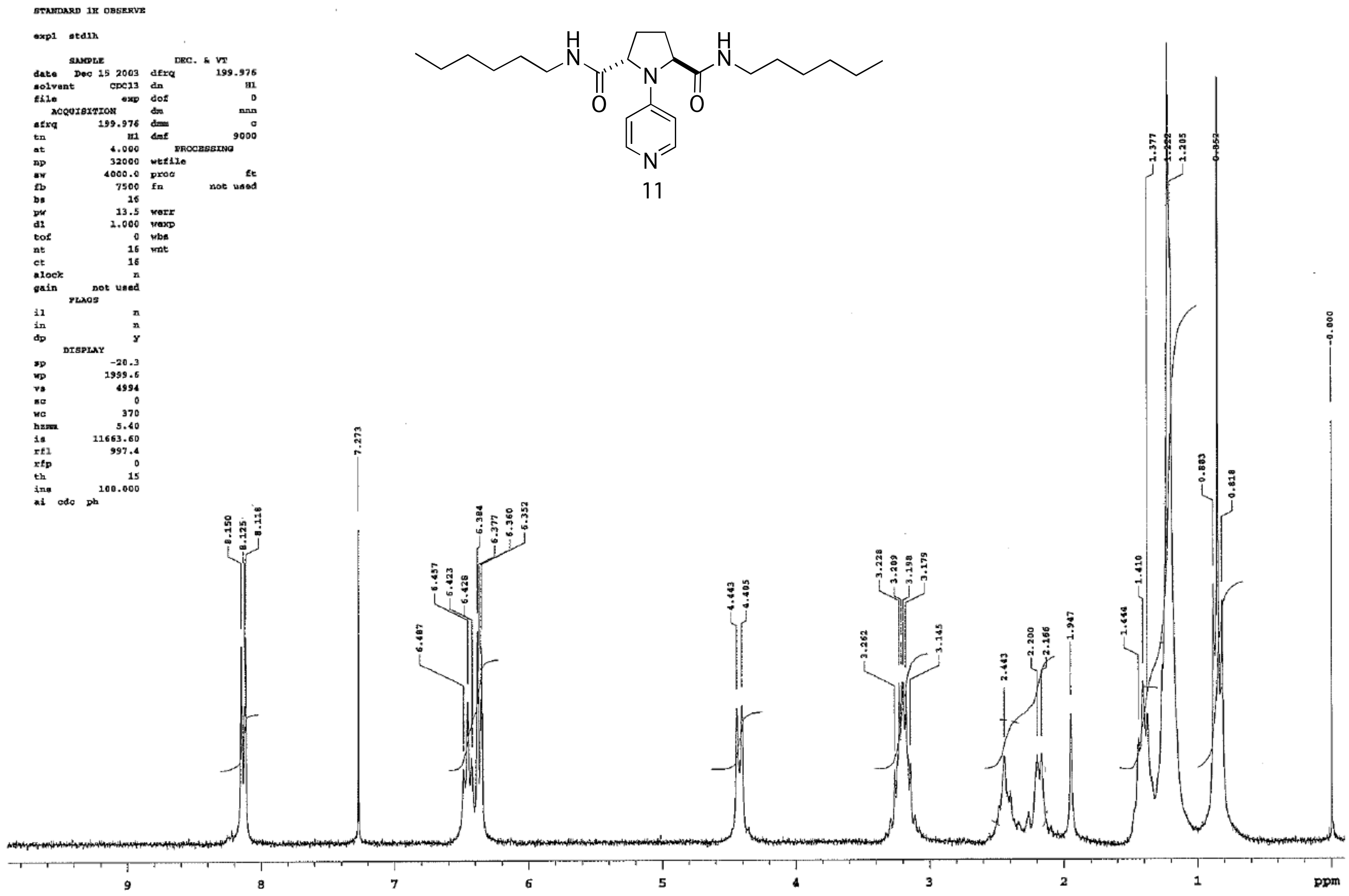
STANDARD 1H OBSERVE
exp1 std1h
SAMPLE
date Dec 15 2003
solvent CDCl3
file exp
ACQUISITION
sfrq 199.976
tn H1
at 4.000
np 32000
sw 4000.0
fb 7500
bs 16
pw 13.5
d1 1.000
tof 0
nt 16
ct 16
alock n
gain not used
FLAGS
il n
in n
dp y
DISPLAY
sp -20.3
wp 1999.6
vs 4994
sc 0
wc 370
hzmm 5.40
is 11663.60
rfl 997.4
rfp 0
th 15
ins 100.000
ai cdc ph
DEC. & VT
dfrq 199.976
dn H1
dof 0
dm nnn
dmm c
dmf 9000
PROCESSING
wtfile
proc ft
fn not used
werr
wexp
wbs
wnt
11
8.150
8.125
8.118
7.273
6.487
6.457
6.423
6.428
6.384
6.377
6.360
6.352
4.443
4.405
3.262
3.228
3.209
3.198
3.179
3.145
2.443
2.200
2.166
1.947
1.444
1.410
1.377
1.222
1.205
0.852
0.883
0.818
0.000
9
8
7
6
5
4
3
2
1
ppm

13C OBSERVE

expl std13c

SAMPLE		DEC. & VT	
date	Dec 15 2003	dfrq	199.976
solvent	CDCl3	dn	H1
file	exp	dof	0
ACQUISITION		dm	yyy
sfrq	50.289	dmm	w
tn	C13	dmf	9000
at	0.999	PROCESSING	
np	29984	lb	1.00
sw	15000.0	wtfile	
fb	7500	proc	ft
bs	32	fn	not used
pw	6.7		
d1	2.000	werr	
tof	0	wexp	
nt	10000	wbs	
ct	1248	wnt	
alock	n		
gain	not used		
FLAGS			
il	n		
in	n		
dp	y		
DISPLAY			
sp	27.0		
wp	10011.9		
vs	6101		
sc	0		
wc	370		
hzmm	27.06		
is	500.00		
rfl	2609.9		
rfp	0		
th	17		
ins	100.000		
ai cdc ph			

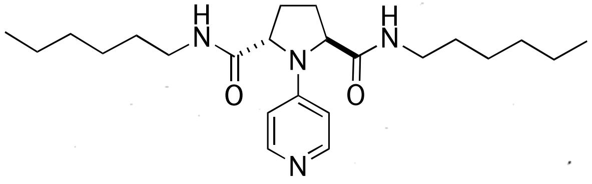

11

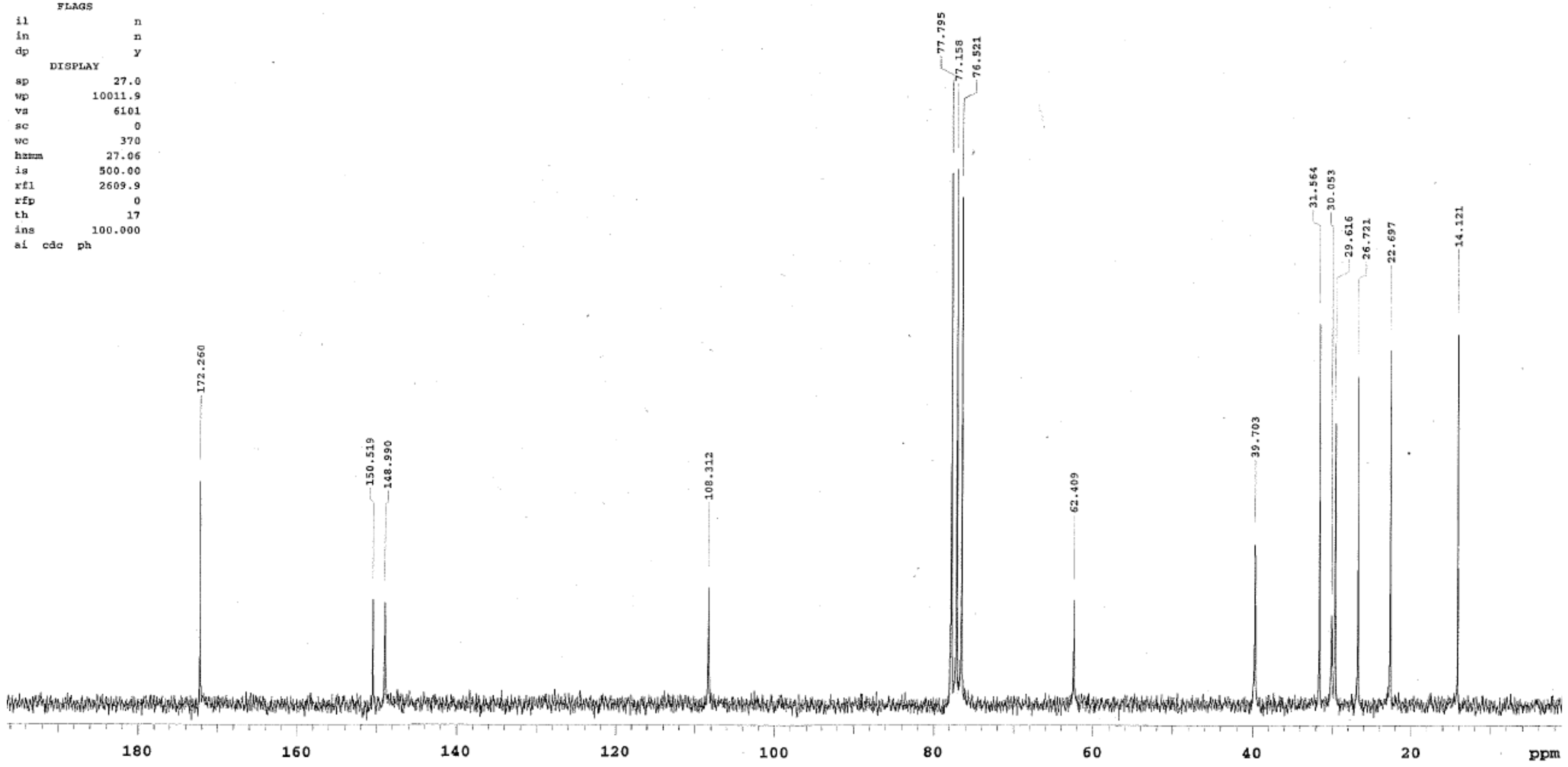

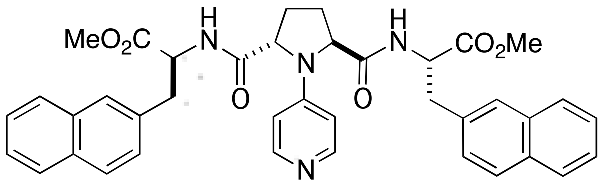

12a

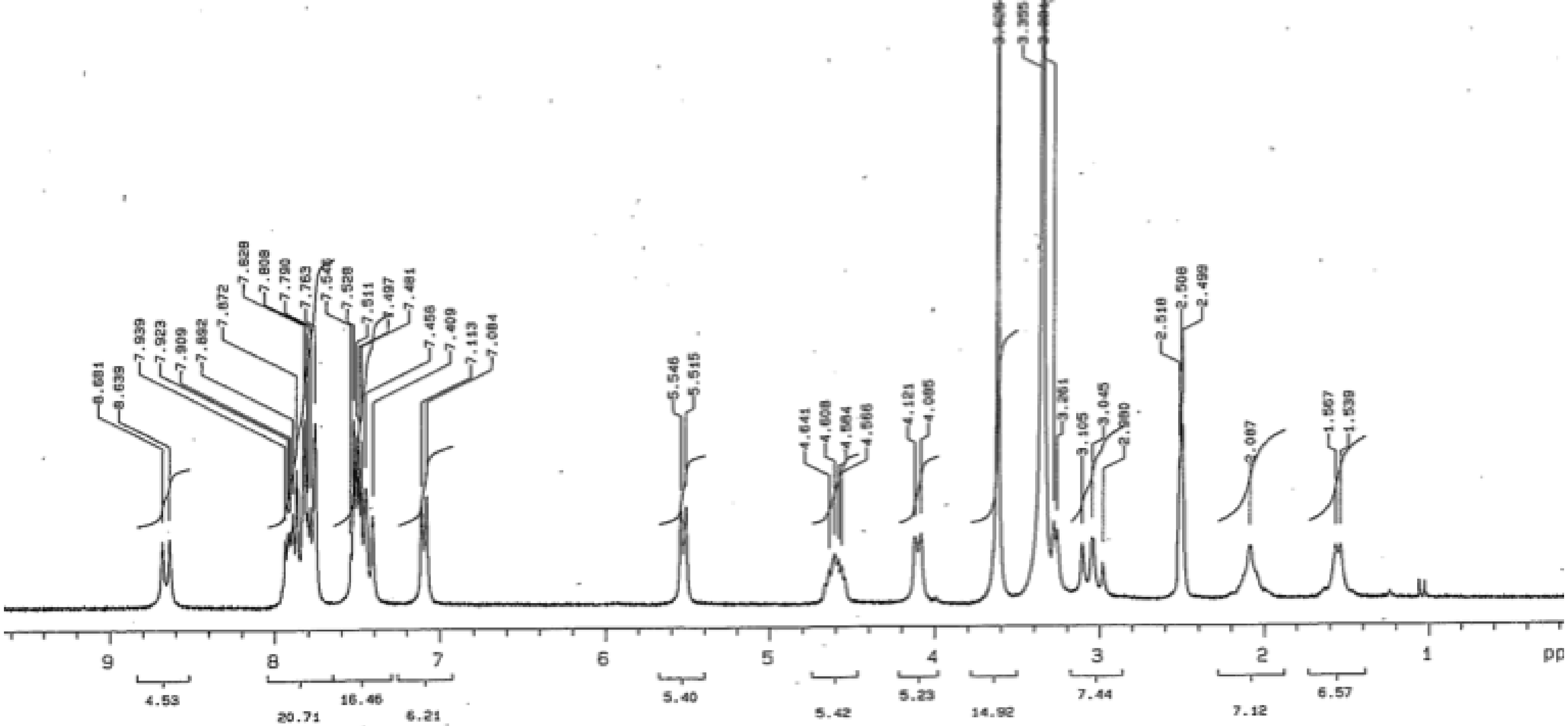

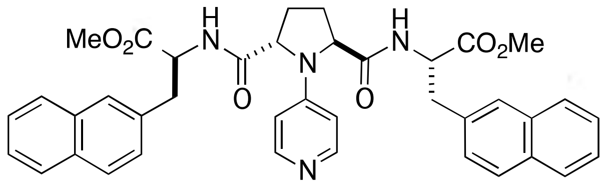
MeO2C
H
N
O
N
N
H
N
O
CO2Me

12a

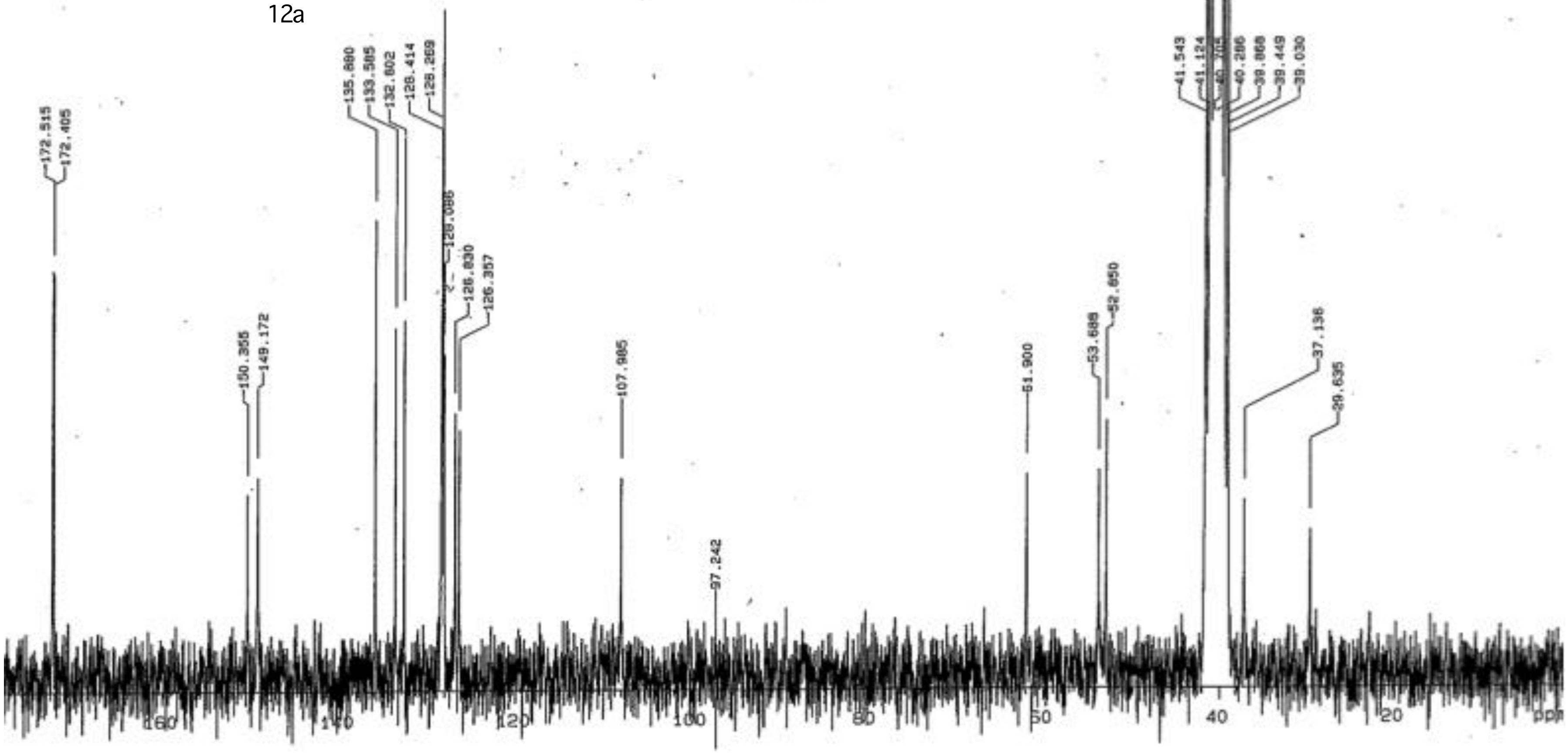
172.515
172.405
150.355
149.172
135.880
133.585
132.802
128.414
128.269
128.086
126.830
126.357
107.985
97.242
61.900
53.688
52.650
41.543
41.124
40.704
40.286
39.868
39.449
39.030
37.136
29.635
160
140
120
100
80
60
40
20
ppm

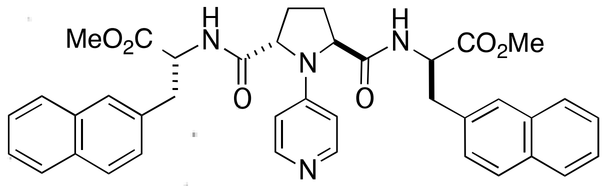
MeO$_2$C
H
N
N
H
N
CO$_2$Me
O
O
N

12b

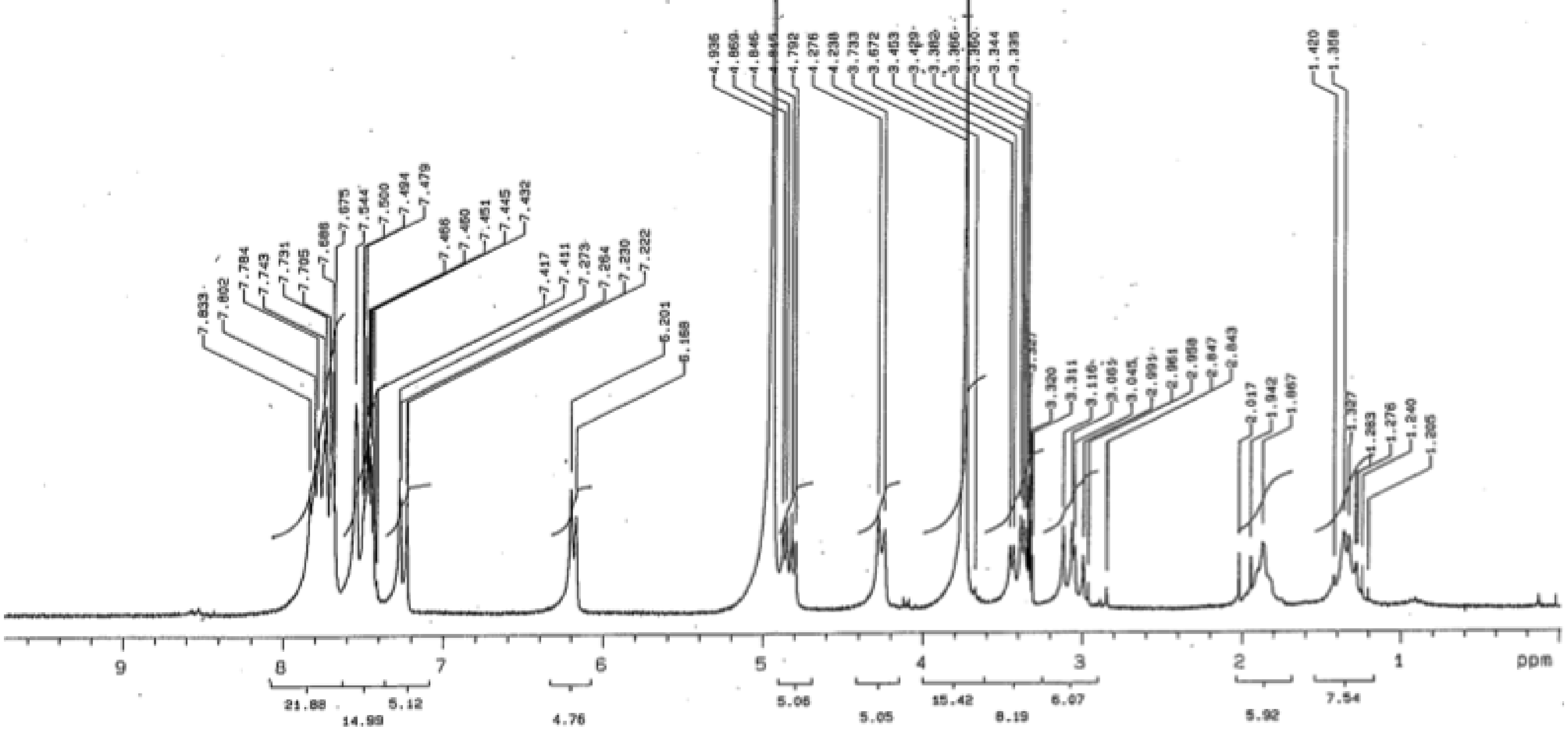
7.833
7.802
7.784
7.743
7.731
7.705
7.688
7.675
7.544
7.500
7.494
7.479
7.466
7.460
7.451
7.445
7.432
7.417
7.411
7.273
7.264
7.230
7.222
6.201
6.188
4.936
4.869
4.846
4.845
4.792
4.276
4.238
3.733
3.672
3.453
3.429
3.382
3.366
3.360
3.344
3.335
3.327
3.320
3.311
3.116
3.085
3.048
2.991
2.961
2.959
2.847
2.843
2.017
1.942
1.867
1.420
1.388
1.327
1.283
1.276
1.240
1.205
9
8
7
6
5
4
3
2
1
ppm
21.88
14.99
5.12
4.76
5.06
5.05
15.42
8.19
6.07
5.92
7.54

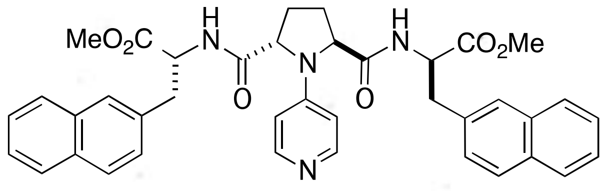

12b

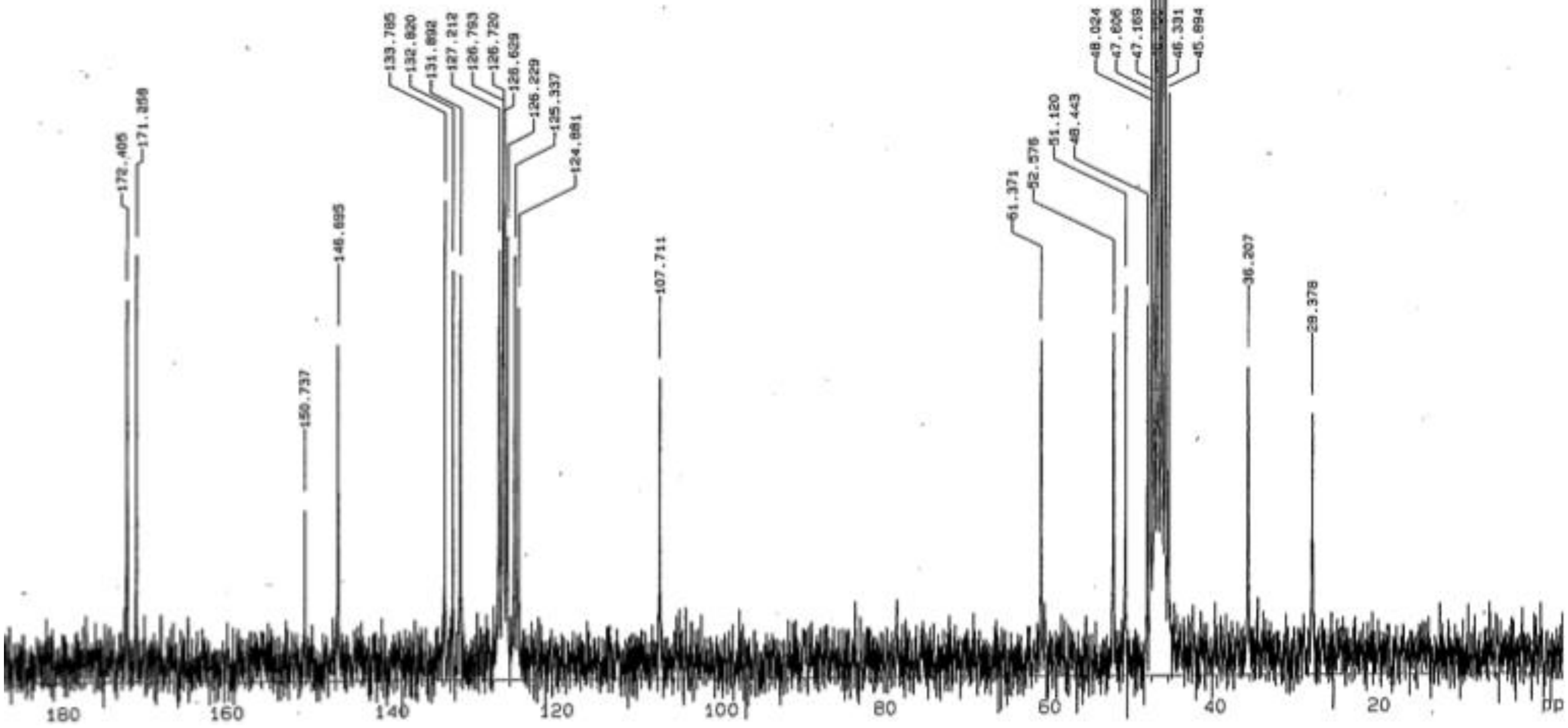

172.405
171.358
150.737
146.895
133.785
132.820
131.892
127.212
126.793
126.720
126.629
126.229
125.337
124.881
107.711
61.371
52.576
51.120
49.443
48.024
47.606
47.189
46.331
45.894
36.207
28.378
180
160
140
120
100
80
60
40
20

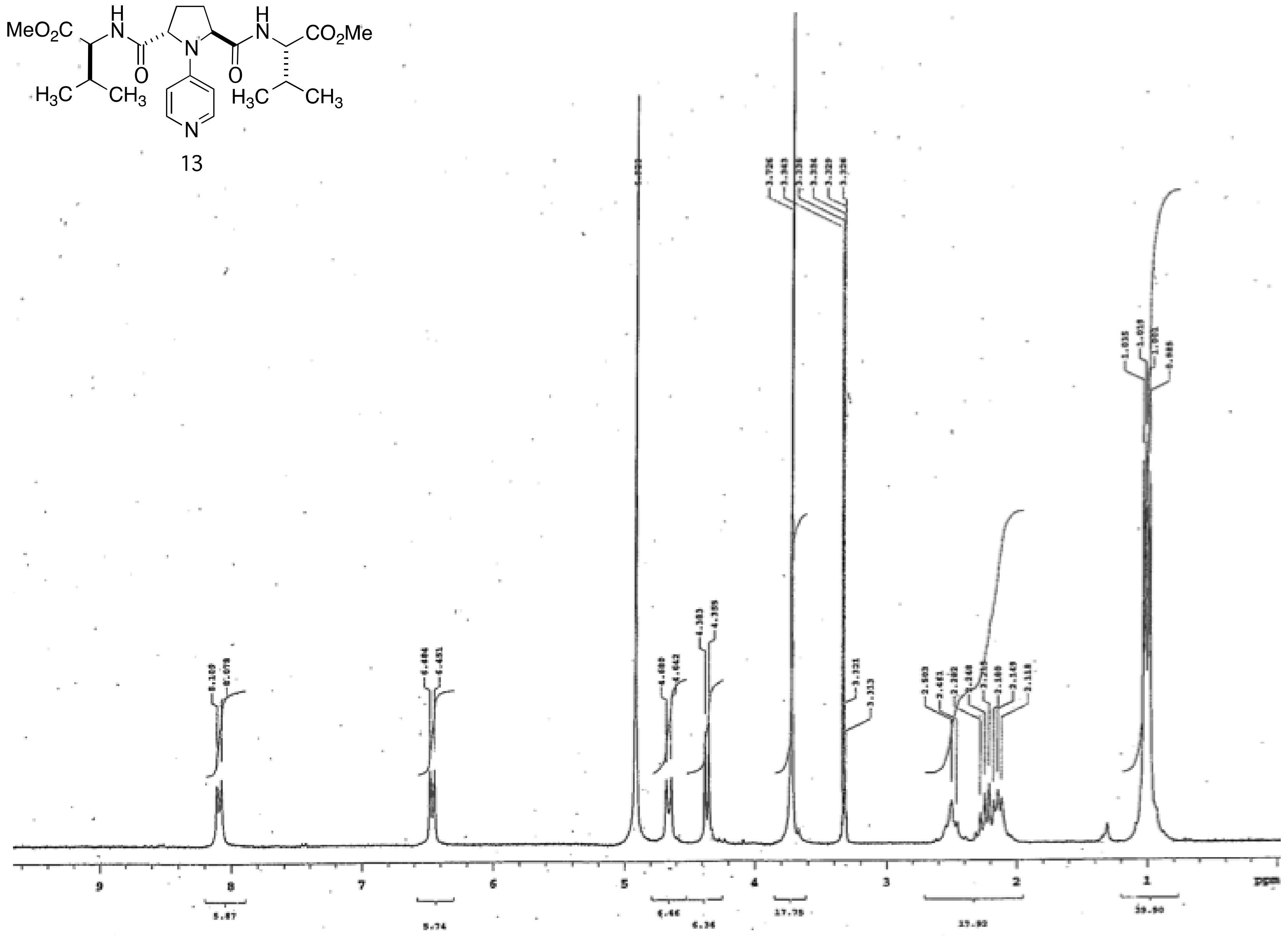
MeO2C
CO2Me
H3C
CH3
H3C
CH3
N
13
9
8
7
6
5
4
3
2
1
ppm

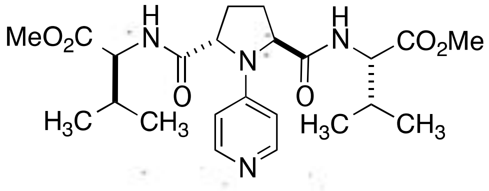

13

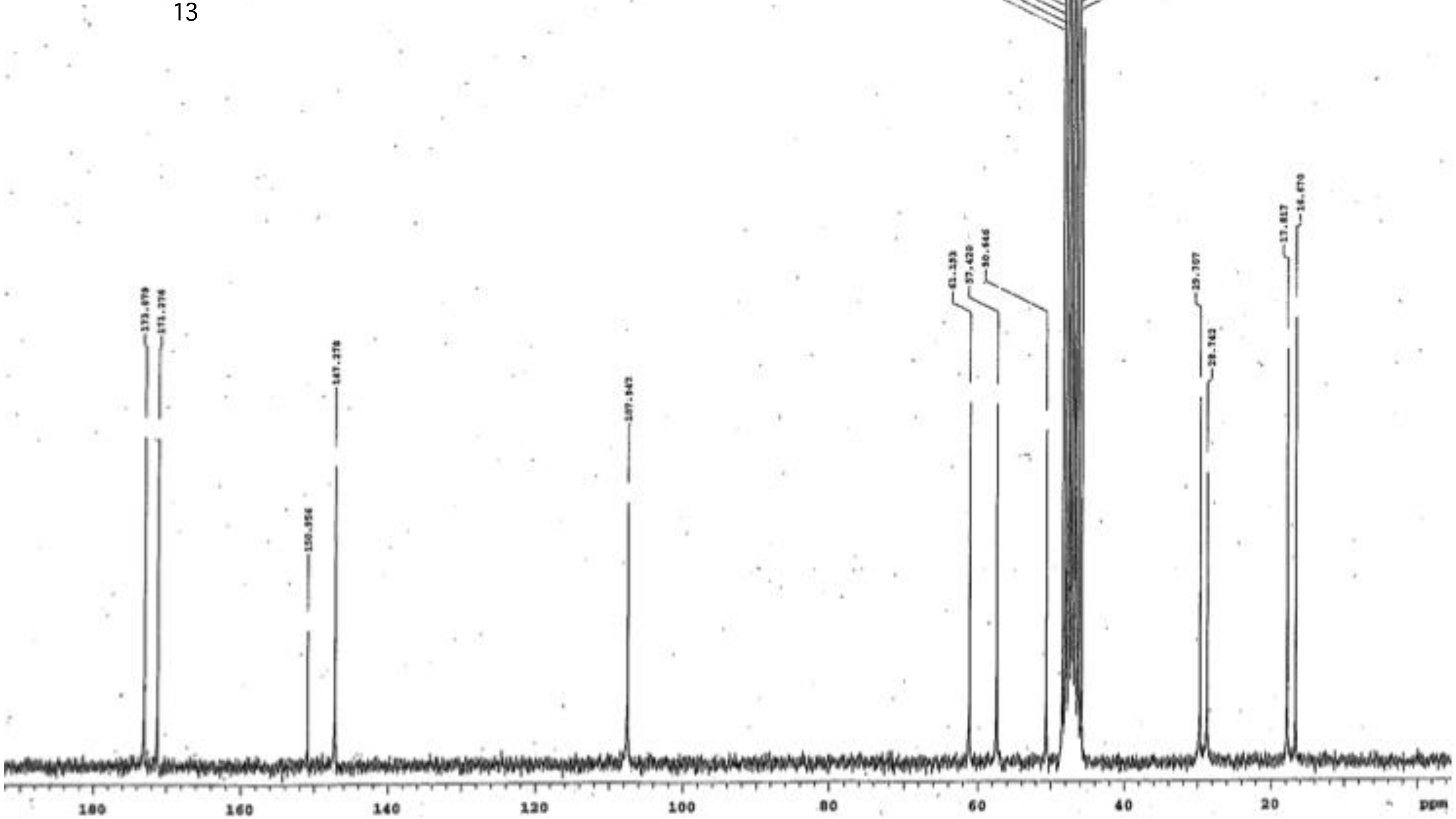

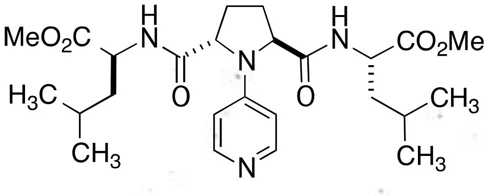

14

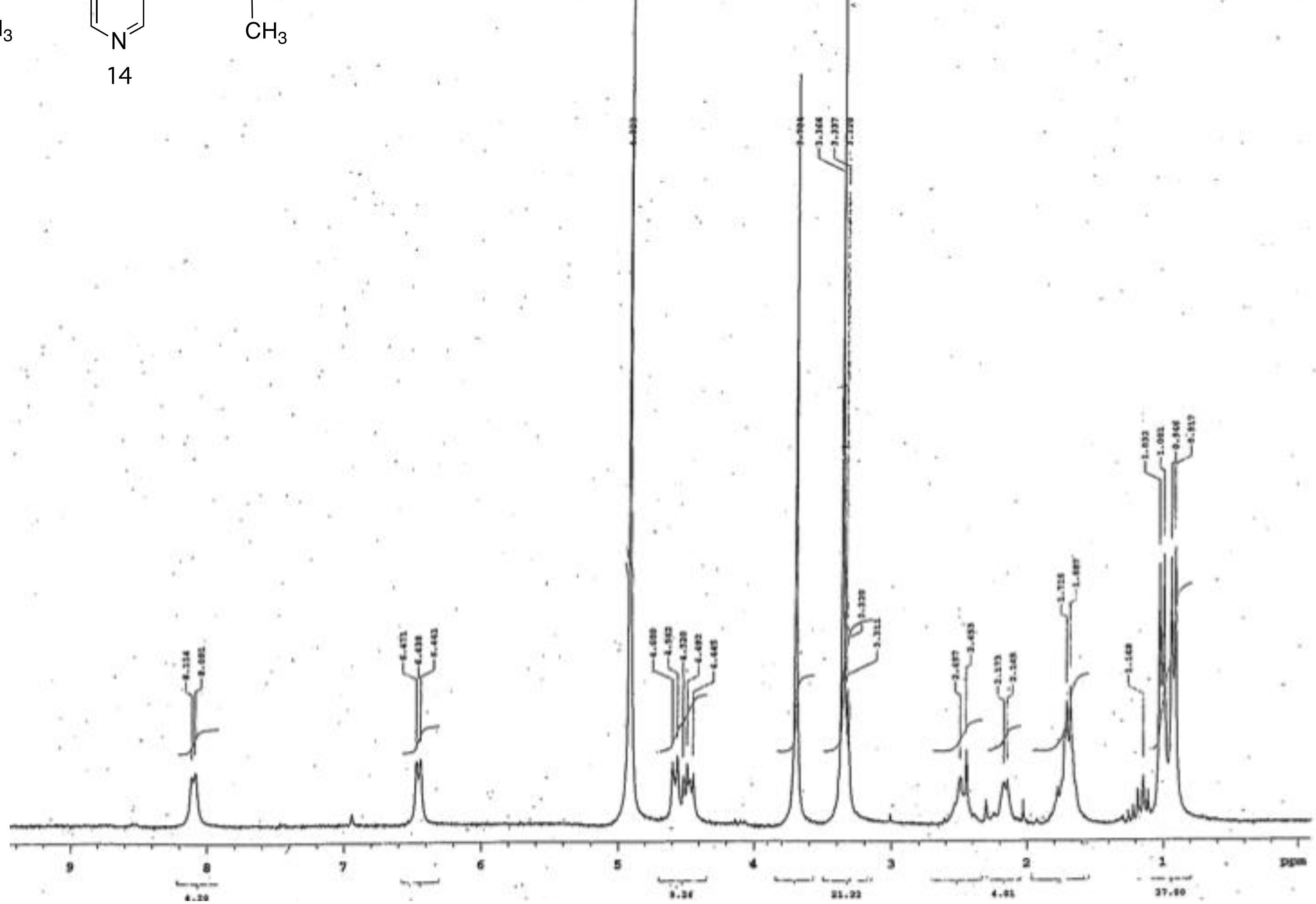

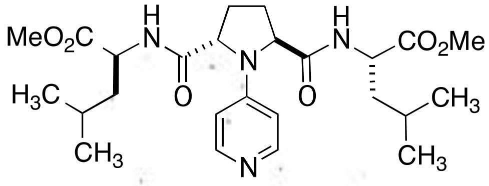
MeO$_2$C
H
N
O
N
N
H
N
O
CO$_2$Me
H$_3$C
CH$_3$
CH$_3$
CH$_3$

14

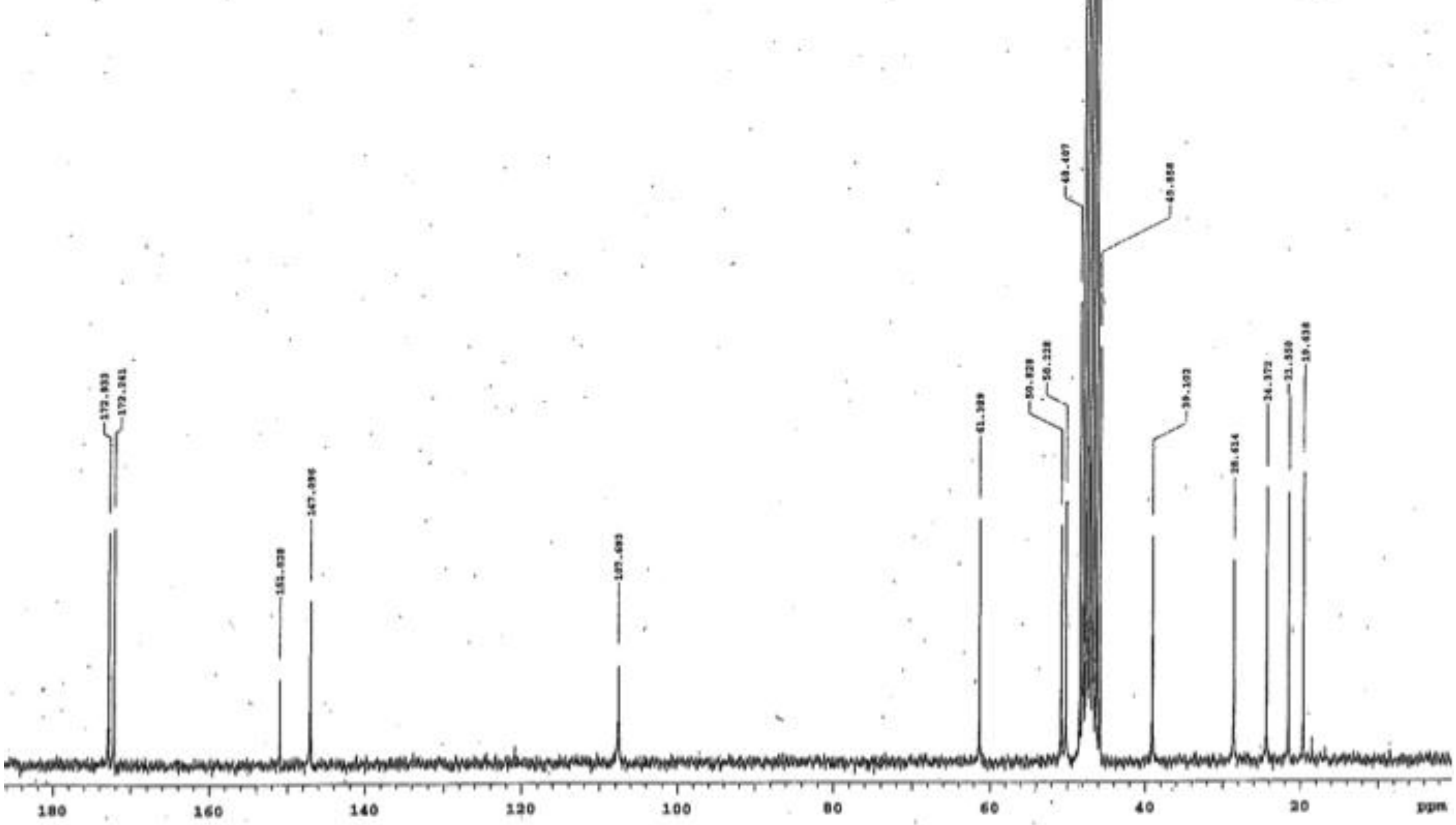
180
160
140
120
100
80
60
40
20
ppm

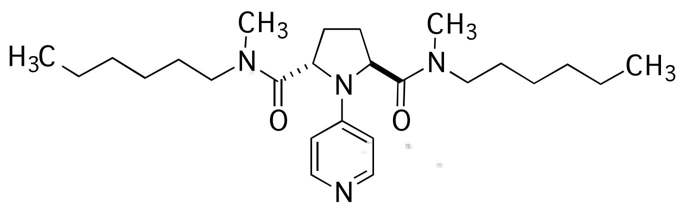

15

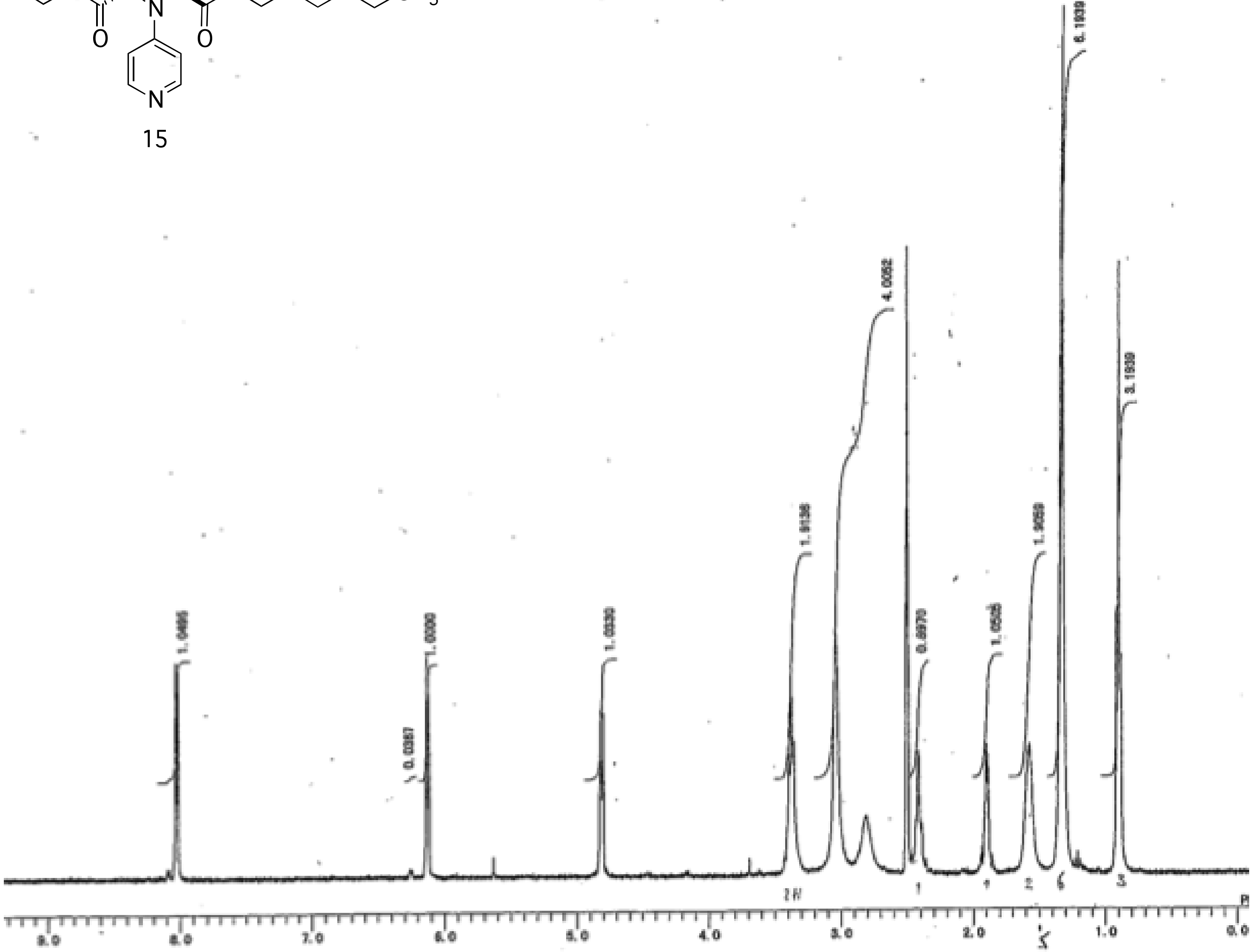

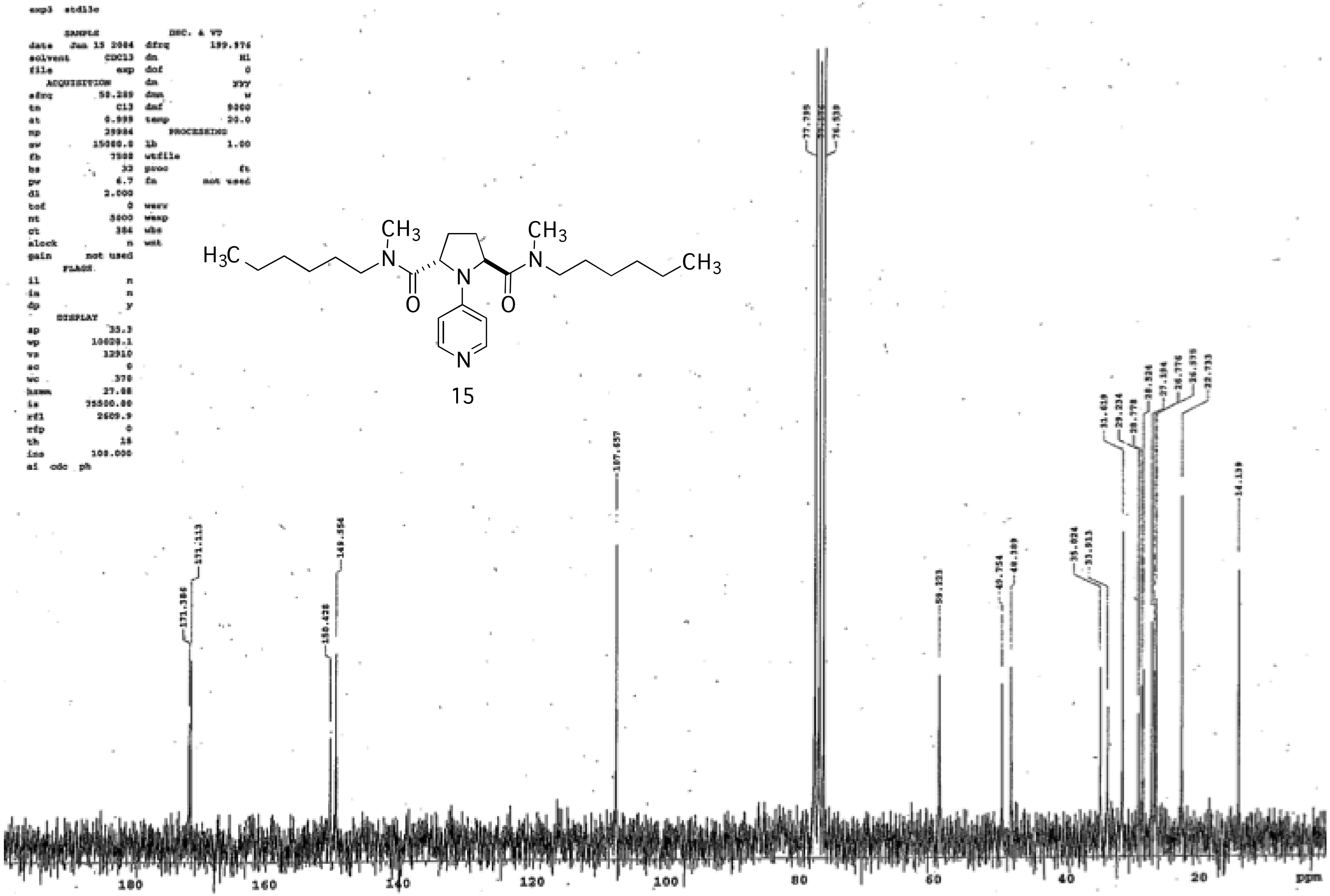

15

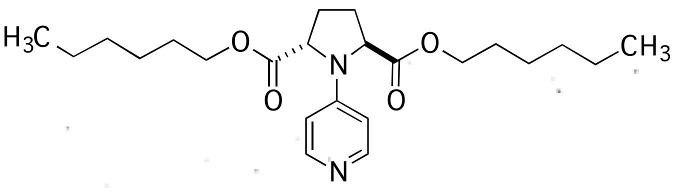

16

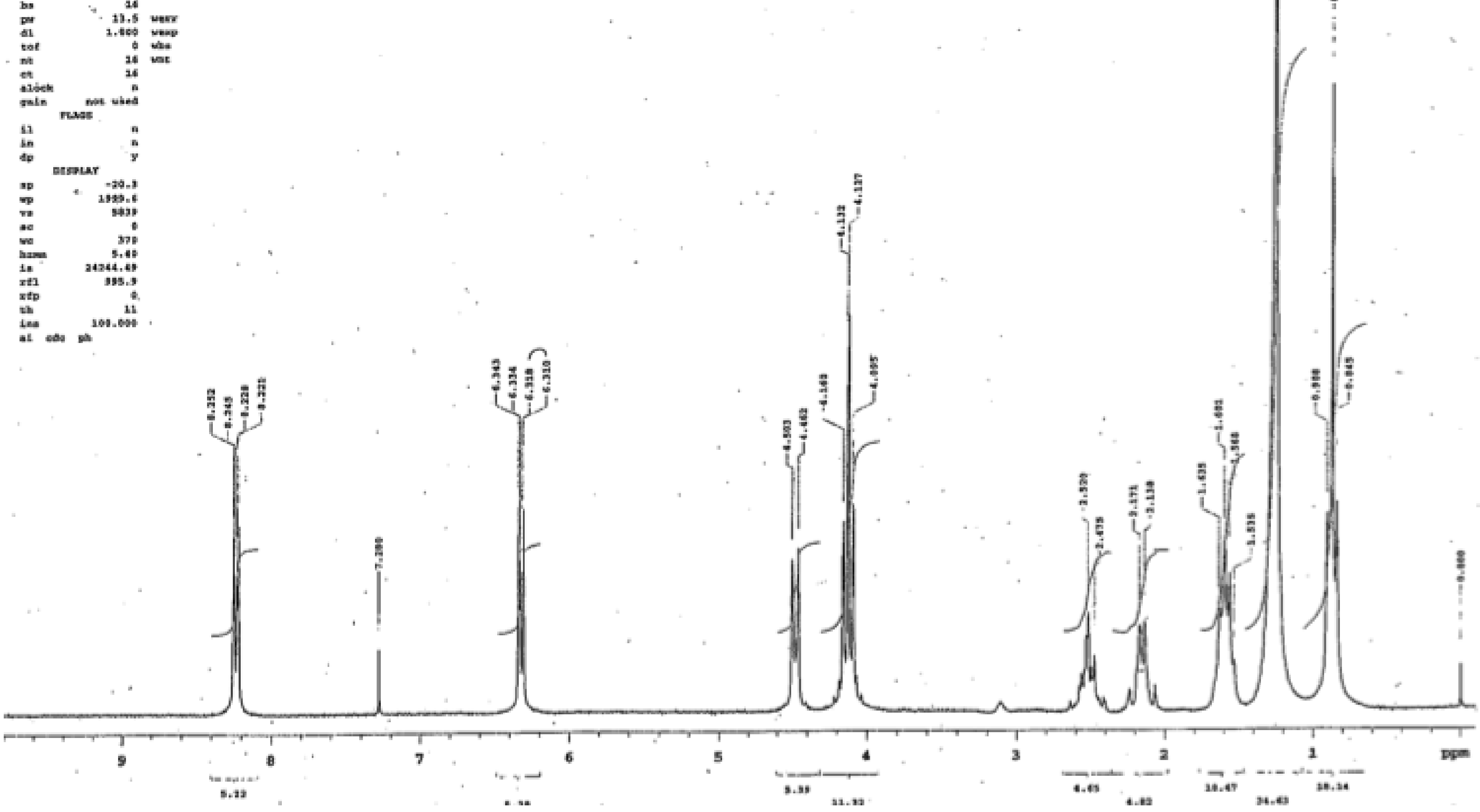

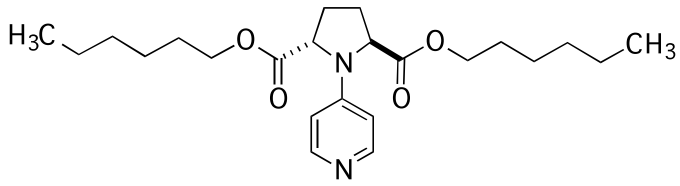

16

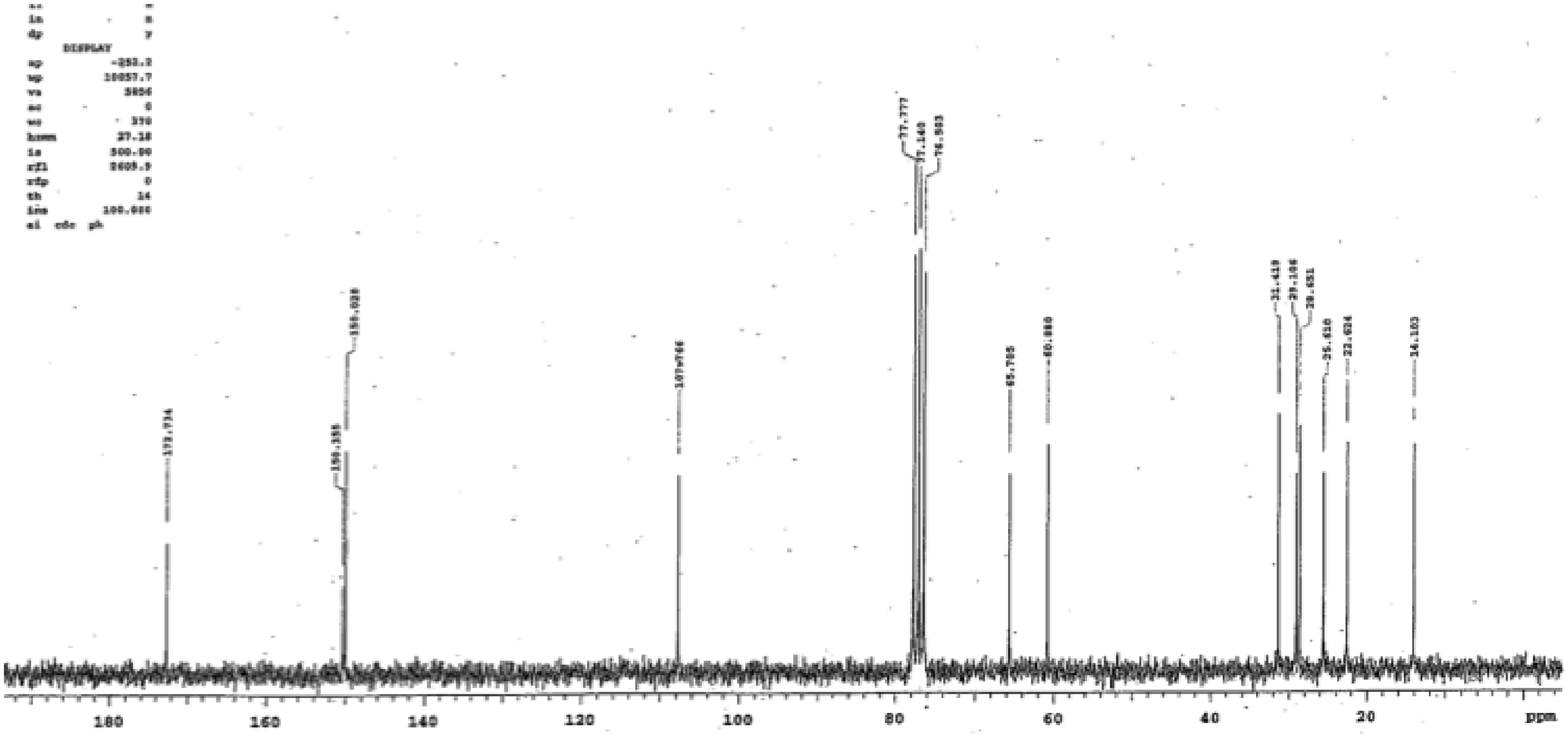

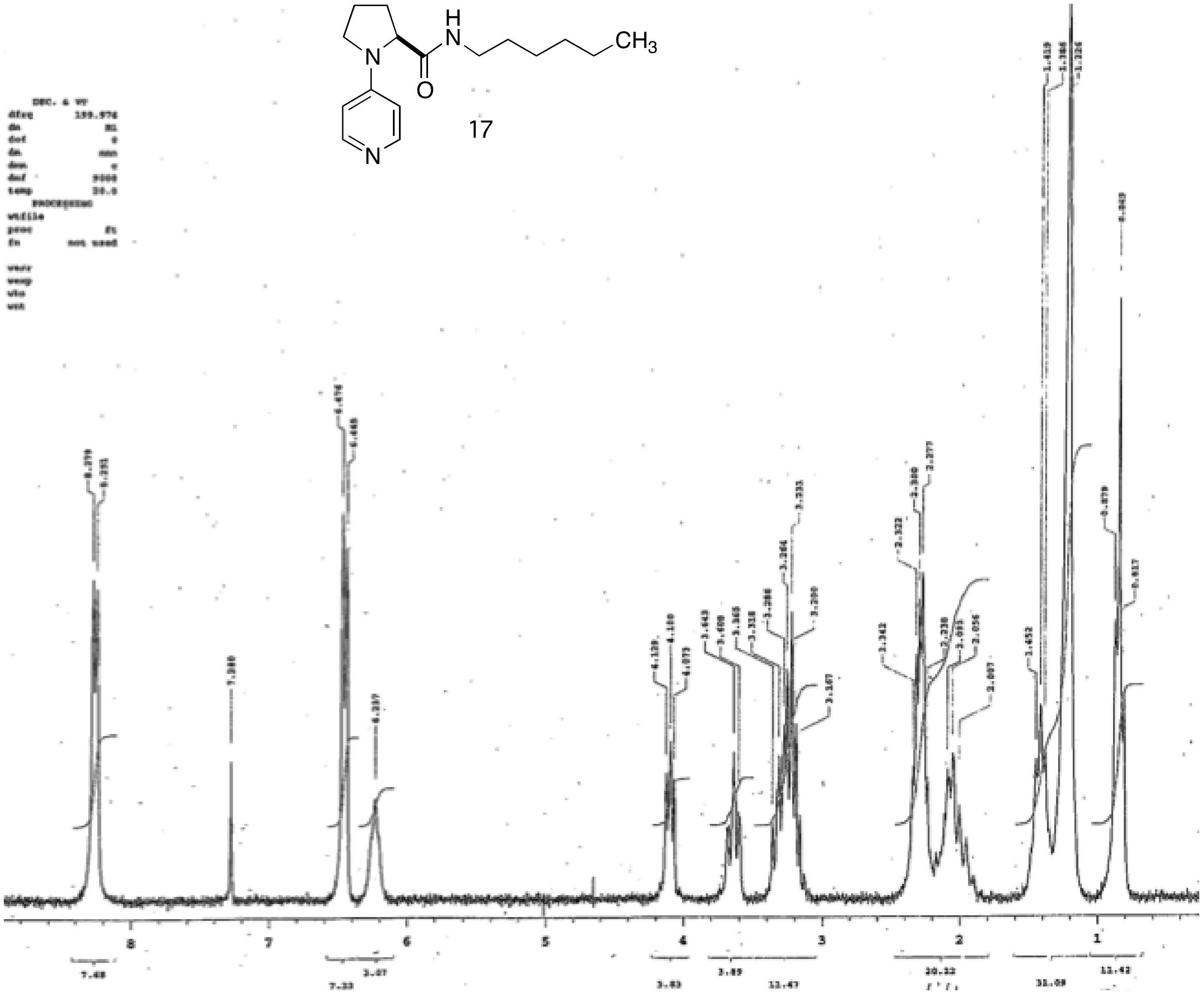
H
N
CH3
N
O
N
17
8
7
6
5
4
3
2
1
7.68
7.33
3.07
3.83
3.89
11.47
20.32
31.09
11.42

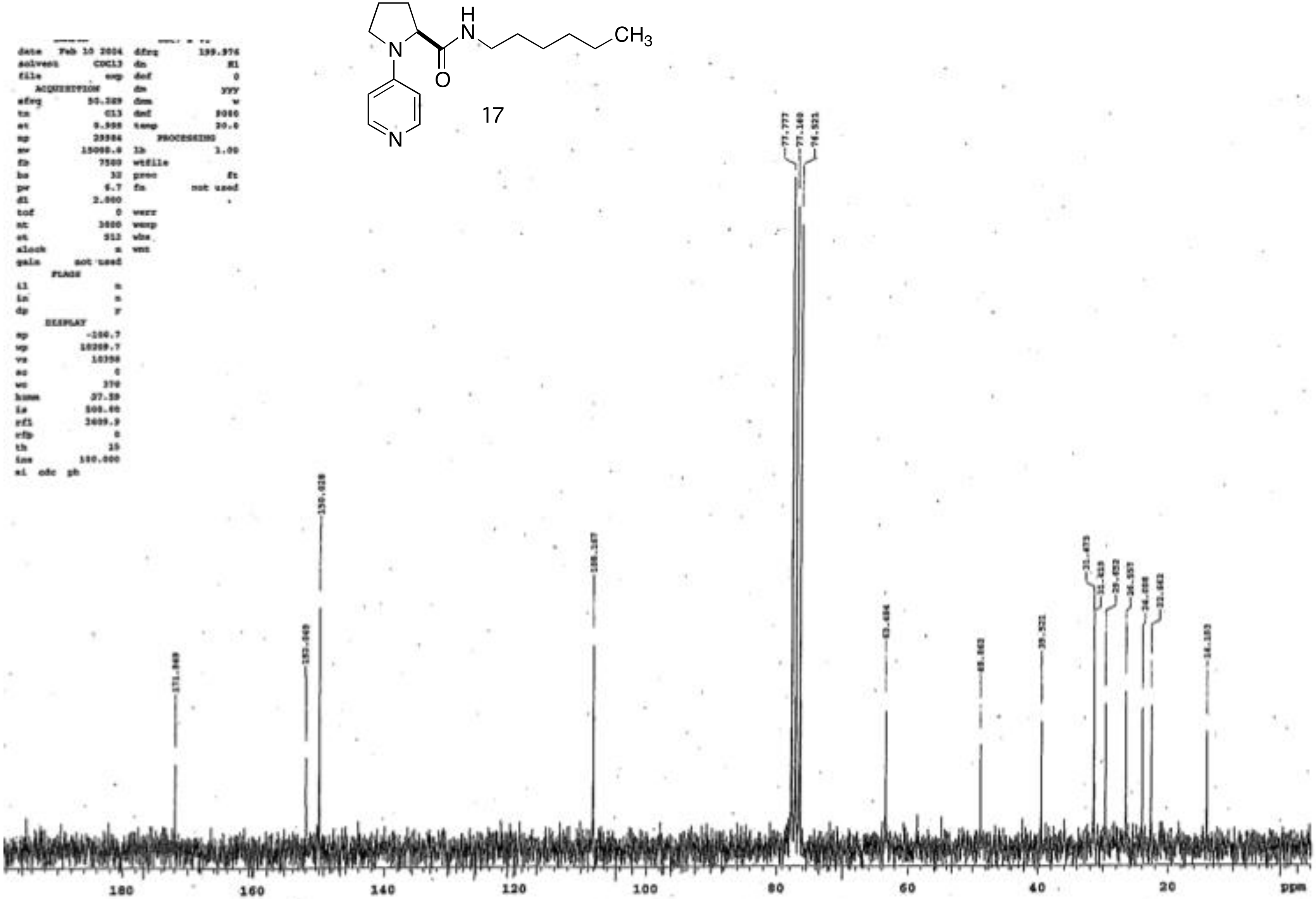

H
N
CH3
O
N
N
17
180
160
140
120
100
80
60
40
20
ppm

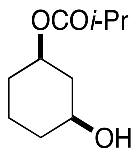

OCO*i*-Pr
OH

20

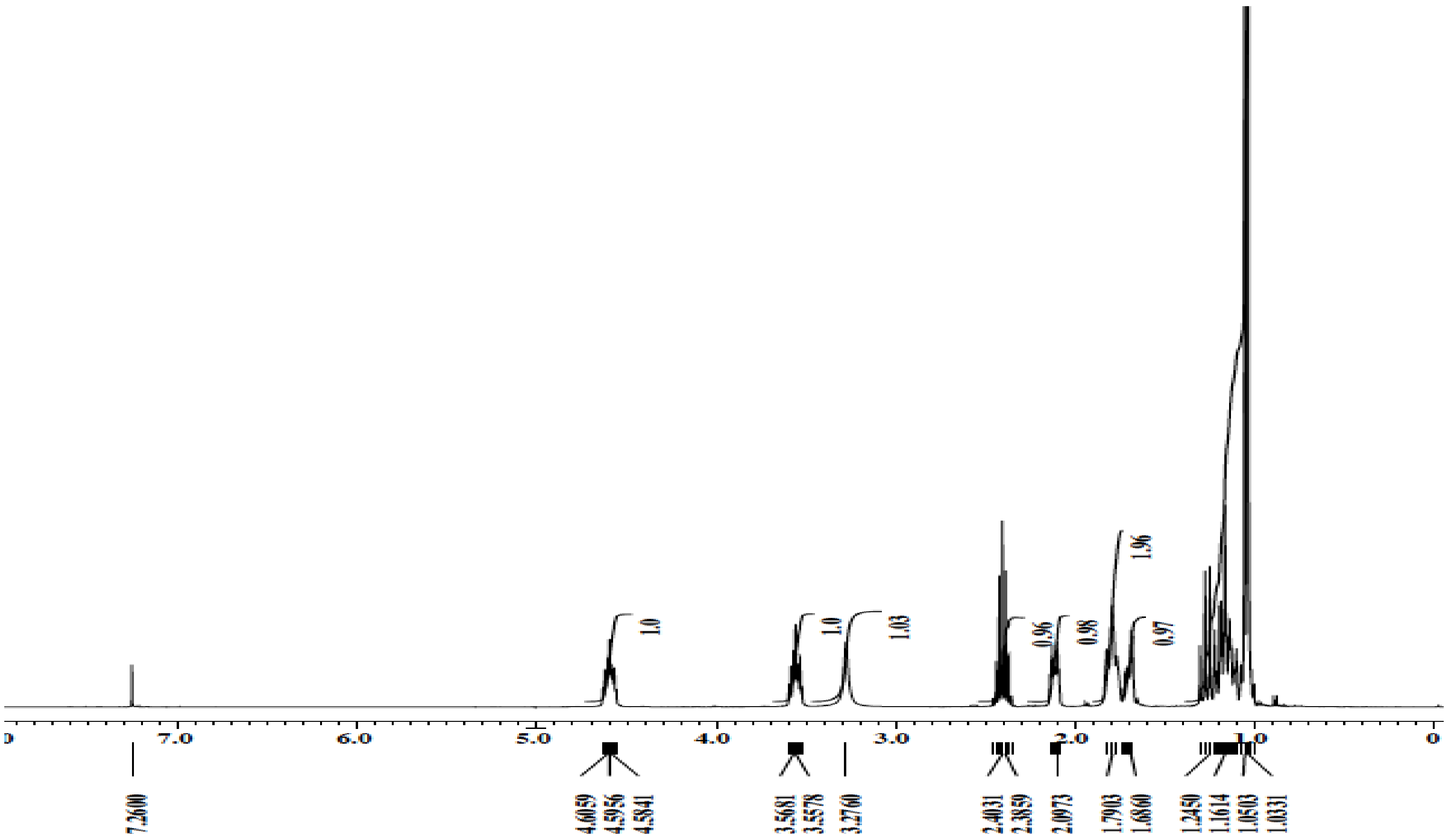

1.0
1.0
1.03
0.96
0.98
1.96
0.97
7.0
6.0
5.0
4.0
3.0
2.0
1.0
0
7.2600
4.6059
4.5956
4.5841
3.5681
3.5578
3.2760
2.4031
2.3859
2.0973
1.7903
1.6860
1.2450
1.1614
1.0503
1.0331

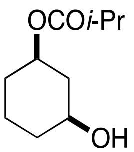

OCOi-Pr
OH

20

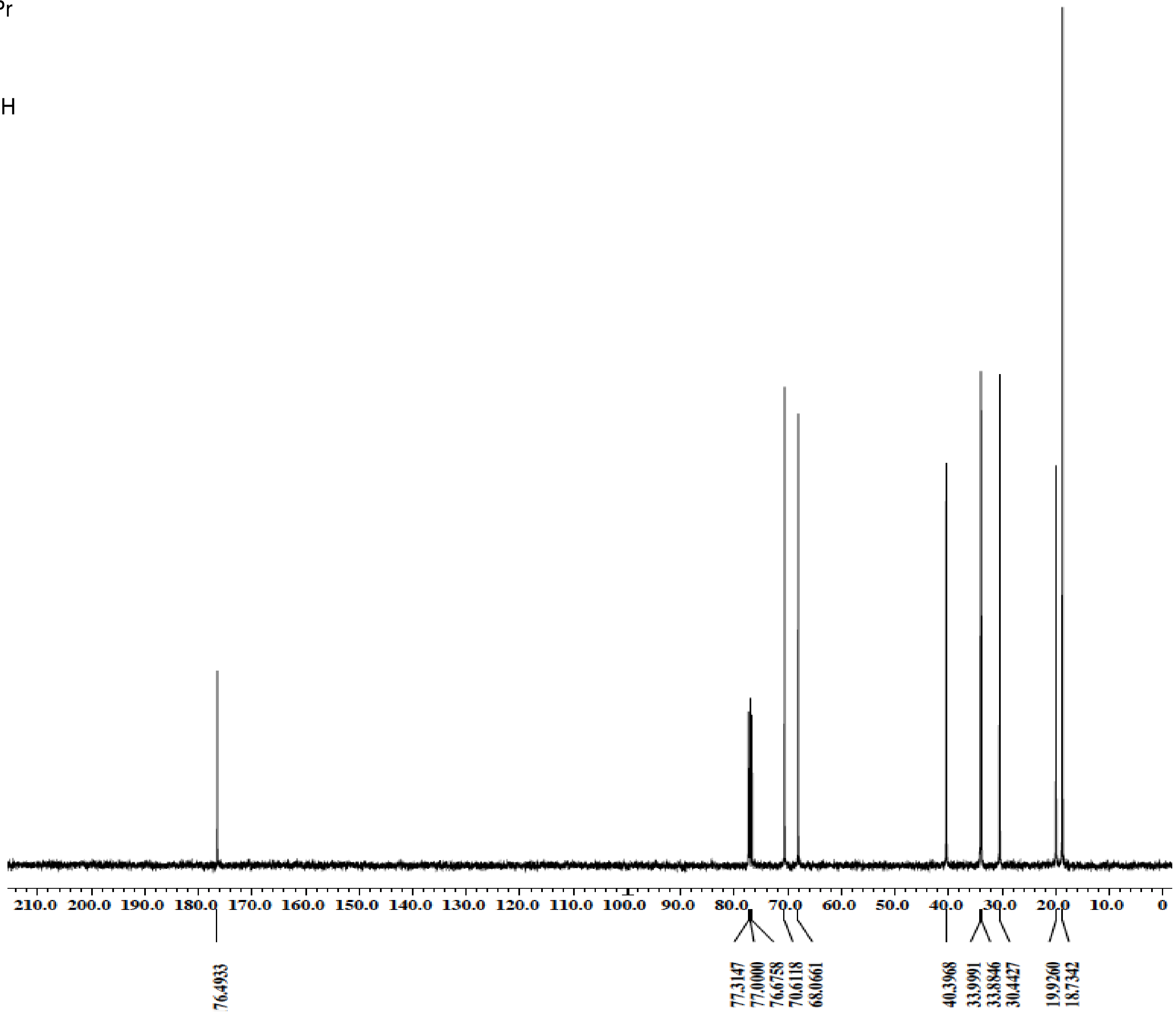

210.0 200.0 190.0 180.0 170.0 160.0 150.0 140.0 130.0 120.0 110.0 100.0 90.0 80.0 70.0 60.0 50.0 40.0 30.0 20.0 10.0 0
76.4933
77.3147
77.0000
76.6758
70.6118
68.0661
40.3968
33.9991
33.8846
30.4427
19.9260
18.7342

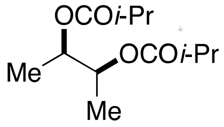
OCO*i*-Pr
OCO*i*-Pr
Me
Me

24a

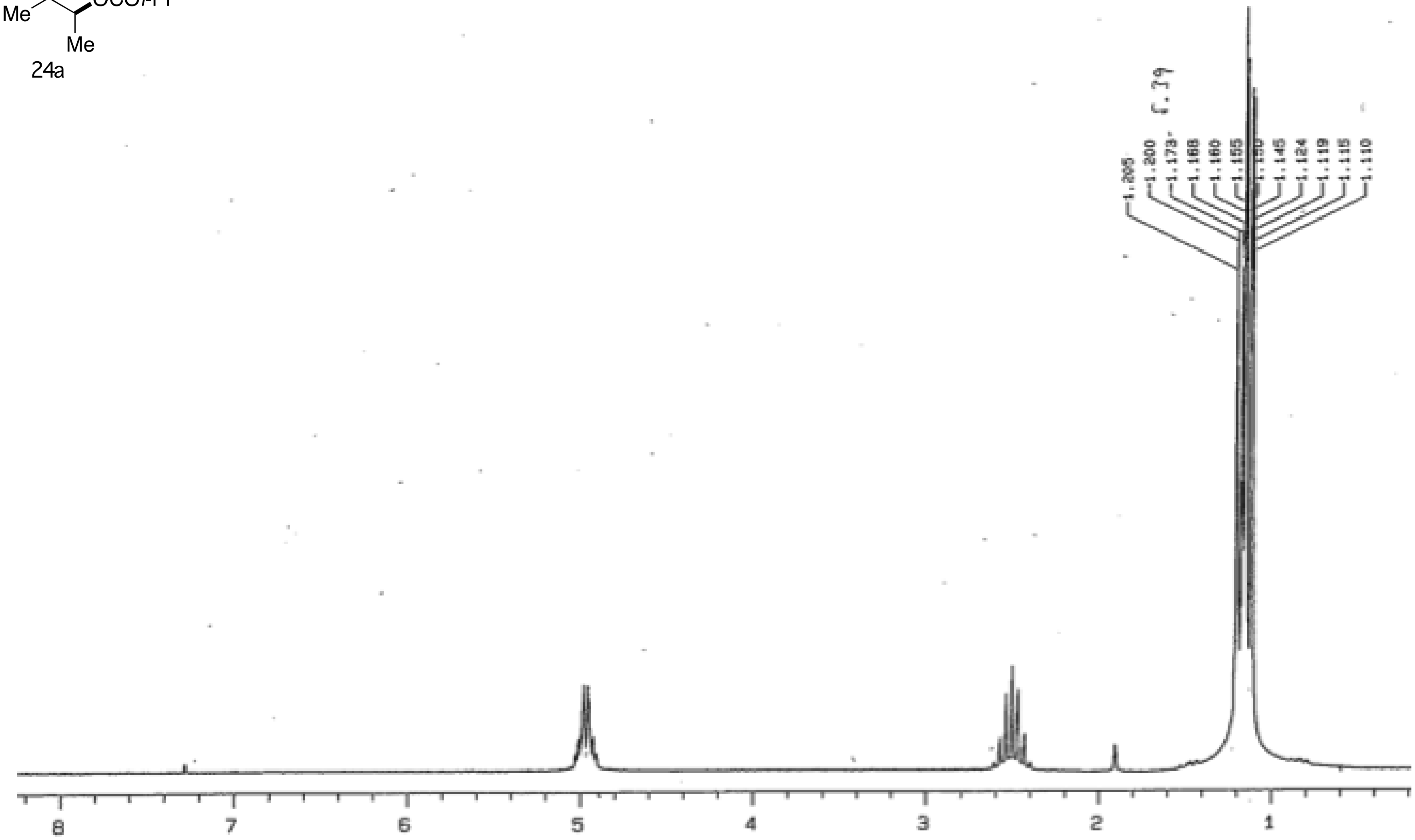
1.205
1.200
1.173
1.168
1.160
1.155
1.145
1.124
1.119
1.115
1.110
8
7
6
5
4
3
2
1

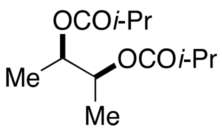

24a

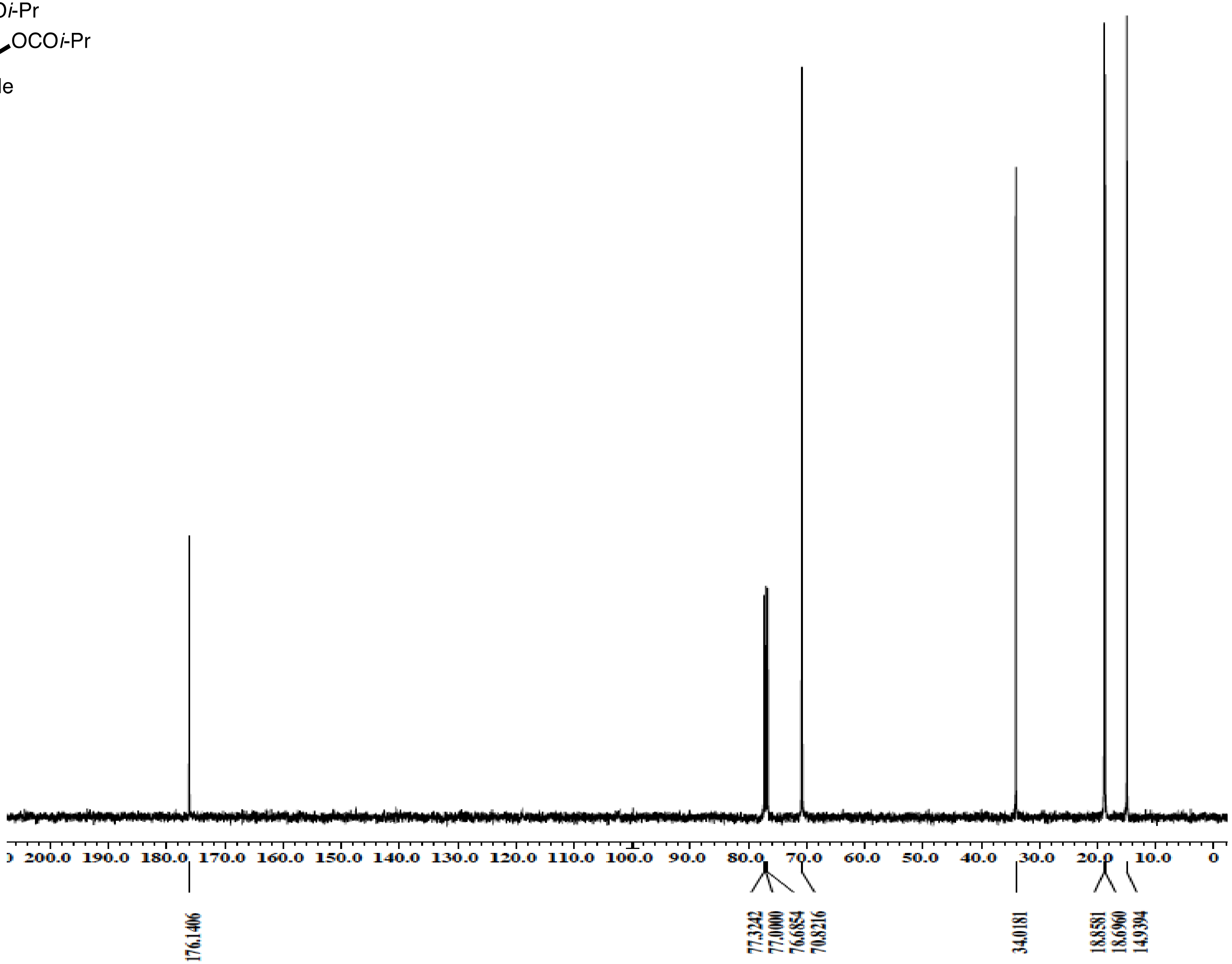
200.0 190.0 180.0 170.0 160.0 150.0 140.0 130.0 120.0 110.0 100.0 90.0 80.0 70.0 60.0 50.0 40.0 30.0 20.0 10.0 0
176.1406
77.3242
77.0000
76.6854
70.8216
34.0181
18.8581
18.6960
14.9394
